# Methods for analysing wildlife DNA methylation data

**DOI:** 10.1093/conphys/coaf091

**Published:** 2026-02-25

**Authors:** Theoni Photopoulou, Ian Durbach, Enrico Pirotta, Ashley Barratclough, Lori H Schwacke, Ryan Takeshita, Gina K Himes Boor, Catriona M Harris, Peter L Tyack, Len Thomas

**Affiliations:** Centre for Research into Ecological and Environmental Modelling, University of St Andrews, The Observatory, Buchanan Gardens, St Andrews, Scotland KY16 9LZ, UK; Centre for Statistics in Ecology, Environment and Conservation, Department of Statistical Sciences, University of Cape Town, Rondebosch 7701, South Africa; Centre for Research into Ecological and Environmental Modelling, University of St Andrews, The Observatory, Buchanan Gardens, St Andrews, Scotland KY16 9LZ, UK; Centre for Statistics in Ecology, Environment and Conservation, Department of Statistical Sciences, University of Cape Town, Rondebosch 7701, South Africa; Centre for Research into Ecological and Environmental Modelling, University of St Andrews, The Observatory, Buchanan Gardens, St Andrews, Scotland KY16 9LZ, UK; National Marine Mammal Foundation, 2240 Shelter Island Drive, Suite 200, San Diego, CA 92106, USA; Marine Mammal Commission, 4340 East-West Highway, Suite 700, Bethesda, MD 20814, USA; National Marine Mammal Foundation, 2240 Shelter Island Drive, Suite 200, San Diego, CA 92106, USA; National Marine Mammal Foundation, 2240 Shelter Island Drive, Suite 200, San Diego, CA 92106, USA; Centre for Research into Ecological and Environmental Modelling, University of St Andrews, The Observatory, Buchanan Gardens, St Andrews, Scotland KY16 9LZ, UK; Sea Mammal Research Unit, Scottish Oceans Institute, School of Biology, University of St Andrews, St Andrews, Scotland KY16 8LB, UK; Centre for Research into Ecological and Environmental Modelling, University of St Andrews, The Observatory, Buchanan Gardens, St Andrews, Scotland KY16 9LZ, UK

**Keywords:** chronological age, DNA methylation, epigenetic age, health, methylation array, wildlife

## Abstract

The analysis of DNA methylation data for wildlife conservation is gaining momentum as the technology for quantifying the methylome becomes mainstream. The use of epigenetic information extracted from tissue samples can be used for estimating chronological age, individual traits and phenotypic variation. Methylation data present an exciting opportunity to study wildlife populations, with the potential to provide insights into age structure, vital rates and health. However, the statistical methodology for answering the emerging research questions has been developed and mostly applied in the human biomedical setting. We review the key methodologies commonly used in wildlife settings, and methods that have been used only in human studies so far that could improve our understanding of wildlife epigenomic changes. We show how the different methods relate to each other and how they link to research questions, illustrating each approach with data from a case study, a large dataset from wild bottlenose dolphins (*Tursiops* spp.) from the US southeast and Gulf coast. Estimating chronological age from models called epigenetic clocks and understanding the relationship between epigenetic indicators of health and exposure to stressors are both key goals in wildlife settings; however, we show that a single model cannot do both accurately. This is a fundamental limitation of clock-type models and might explain why some age-related health conditions have been found to be related to epigenetic age and others not. Decoupling the analysis of age and health is challenging because the two are confounded but is especially important in wildlife settings where age prediction is often the main analytical objective.

## Introduction

The statistical analysis of epigenetic modifications to the genome of wild animals presents an opportunity to estimate individual traits and understand individual health, where health is defined as the ability of an organism to adapt to and manage threats to survival and reproduction (Tyack *et al.,* 2022). Health has become a central concept in wildlife demography and conservation because health metrics integrate the effect of stressors on individuals. For many wildlife species, especially large vertebrates, making population-level inferences about vital rates or disease status is often limited by the small sample sizes associated with physical examinations in the field. Assessing epigenetic data, such as DNA methylation, from tissue samples offers an appealing alternative for ecology and conservation. Cells can regulate gene expression using methylation modifications at cytosine–phosphate–guanine (CpG) sites across the genome—collectively called the methylome. Changes in DNA methylation (DNAm) may result from developmental processes during aging, exposure to environmental stressors, or from changes in health, leading to expressions of different phenotypes. In addition to functional and/or adaptive changes, shifts in DNAm patterns also happen as the result of random changes that accumulate over time as individuals age (humans (Horvath and Raj, 2018), animal populations (Husby, 2022; Balard *et al.,* 2024), though neither process is well understood. Accumulated changes in DNAm have been used as an indicator for long-term health in humans. Here, we explore, from a methodological perspective, the use of DNAm in wildlife to predict age, and as an indicator of exposure to stressors, overall health and specific health conditions. A variety of methylation arrays and next-generation sequencing approaches are available to measure the methylation status at tens or hundreds of thousands of CpG sites (Polanowski *et al.,* 2014; Arneson *et al.,* 2022), which is typically reported as the proportion of methylation (at each unique site) in the population of cells that make up the sample. The enormous potential of epigenetics to improve our understanding of wildlife populations and individual health makes it important to evaluate the statistical methods for quantifying changes in DNAm. These have mostly been developed and applied in the human biomedical setting.

Methylation data are relatively new and methods for their analysis face a variety of statistical challenges (Teschendorff and Zheng, 2017; Teschendorff and Relton, 2018; Bell *et al.,* 2019; Yousefi *et al.,* 2022). The large number of CpG sites measured for a single sample make this a “small $n$, large $p$” problem, where the measured variables ``${p}$'' greatly outnumber the individual samples “$n$” represented in the data. CpG sites in close proximity to one another tend to share a methylation status (either both methylated or both unmethylated) making the data highly correlated; the likelihood of this “co-methylation” (Affinito *et al.,* 2020) decreases with increasing distance between the sites (typically on the same chromosome, but potentially based on chromatin configuration across chromosomes). Genes that rely on similar transcription factors or other regulatory elements can also have highly correlated DNAm patterns across their CpG sites (Yao *et al.,* 2015). Statistical tests must have adequate power to detect modest changes or changes that occur in a relatively small proportion of the population. There are many sources of both human error and technical noise, some of which are practically challenging to investigate. There are also many potential confounding variables, which may be unobserved, and whose presence may bias parameter estimates and, depending on the research question, introduce spurious associations. For example, methylation is affected by disease status, it can vary between tissue types, and it can also vary within a single tissue sample (Field *et al.,* 2018; Forest *et al.,* 2018). Studies of wild animal populations face the additional challenges of smaller sample sizes than human studies, and potentially unknown or imprecisely known age of individual animals (Newediuk *et al.,* 2025). Although DNAm data can be used to estimate age, known-age individuals are required for initial model development and validation. Analysts must make pragmatic decisions about which of these challenges to address and which to ignore or simplify by assumption, e.g. of error-free measurement. The direction of causality in the process of methylation is also important to consider when formulating a research question and analytical approach. If changes in DNAm are assumed to be random then there is no need to model causality. If changes in DNAm are the result of underlying cellular processes in response to stressors or disease, then causality can go both ways since these factors can also alter the methylation process directly. From an analytical perspective, DNAm data have often been used to explain phenotypic variability, such as disease status, or used to predict age, but can equally validly be treated as a response variable.

There are many reviews of statistical methods for the analysis of methylation data, such as (Teschendorff and Zheng, 2017; Teschendorff and Relton, 2018; Bell *et al.,* 2019) and (Yousefi *et al.,* 2022). This paper builds on that work, framing it from the perspective of the study of wild animal populations (see also (Newediuk *et al.,* 2025)) and focusing on methods using health as a response or predictor. Our aim is not to provide an instructional guide, such as Laine *et al.* (2023); instead, we present an organized summary of the methods from the human literature that have been or could be used in the wildlife setting to improve our understanding of wildlife epigenomic changes through statistical inference. Such a review is of interest because (i) studies of wild animal populations are fundamentally different to those of human populations, and drawing out the methodological implications of these differences provides insights for both fields; (ii) methods development stems nearly entirely from the human literature, and recent developments in the human literature provide a potential roadmap for future research on animal populations and a warning of hidden obstacles and (iii) statistical themes and considerations around the issues raised above warrant further discussion within the wildlife context.

First, we summarize the applications for which wildlife DNAm data have been analysed to provide a high-level overview of the field of wildlife epigenetics at this point. We provide a concise summary of how the data can be generated for mammals using a commonly used custom mammalian methylation array (HorvathMammalMethylChip40) with 37 492 CpG sites (Arneson *et al.,* 2022), followed by a review of the methods used for analyzing wildlife DNAm data. Although our review focuses on methylation array data, Reduced Representation Bisulfite Sequencing (RRBS) offers an alternative approach for generating DNA methylation data in wildlife. Unlike arrays, RRBS can detect methylation at any CpG within the sequenced regions, providing base-pair resolution beyond the predefined probe sites included on array platforms (Fennell *et al.,* 2022). This flexibility makes it especially valuable for non-model species lacking array resources, though it comes with limited comparability across studies, dependence on genome quality and greater analytical complexity, including sparse matrices, overdispersed counts and detection uncertainty (Shafi *et al.,* 2017). The remainder of the paper is then organized along two main lines: methods that treat DNAm as a response and methods that treat DNAm as a predictor (of age or health, although relevant to any phenotype). We focus on the analysis of processed methylation data, omitting other ways of generating DNAm data and associated pre-processing steps, e.g. filtering, normalization, batch correction techniques (see (Wilhelm-Benartzi *et al.,* 2013, Müller *et al.,* 2019)) as well as downstream analyses of relevant CpG sites, e.g. chromatin state analysis, functional enrichment analysis [see (Campagna *et al.,* 2021)]. We illustrate methods and relevant statistical issues using a case study where we analyse DNAm data from a sample of 476 bottlenose dolphins (including Tamanend’s (*Tursiops erebennus*) and common (*Tursiops truncatus truncatus*) bottlenose dolphins) from the USA Southeastern and Gulf coasts (Barratclough *et al.,* 2024). We close with a discussion of the main differences between epigenetic studies of human and wildlife populations, and of what we see as three key statistical considerations facing wildlife studies: decoupling age and health prediction, informed choices of baselines for health assessments, and clarity about study objectives.

## Applications of DNAm in wildlife studies

Epigenetic research in wild animal populations has provided insights into how DNA methylation influences various biological outcomes, including phenotypic plasticity, adaptation, development, aging and responses to environmental stressors (Husby, 2022; Balard *et al.,* 2024). This is often done using Epigenome-Wide Association Studies (EWAS) (Campagna *et al.,* 2021), where the aim is to identify differences in epigenetic markers (e.g. CpG sites) between groups or along a gradient (e.g. age). A diverse range of taxa has been studied, encompassing mammals, birds, fish, reptiles and invertebrates, highlighting the pervasive effects of epigenetic mechanisms in natural settings. As our focus is on methodological developments, we give only a brief summary of this literature here (for more comprehensive reviews, see (Bossdorf *et al.,* 2008, Danchin *et al.,* 2011, Hu and Barrett, 2017, Lea *et al.,* 2017, Husby, 2022, Sadler, 2023, Balard *et al.,* 2024)).

One stream of research has examined how DNA methylation contributes to phenotypic plasticity in the wild. Methylation patterns have been linked to differences in environmental salinity in three-spined sticklebacks (Artemov *et al.,* 2017; Heckwolf *et al.,* 2020), to beak morphology and other phenotypic traits in urban and rural populations of Darwin’s finches (McNew *et al.,* 2017), and to traits such as immune function in wild house sparrows (Hanson *et al.,* 2021). These links suggest that epigenetic mechanisms may buffer populations against environmental variation.

DNA methylation has also been widely explored as a marker of aging in wild animals. Epigenetic clocks—models that predict chronological age from methylation at specific sites—have been developed for many taxa (Zoller and Horvath, 2024), including but not limited to marine mammals (e.g. bottlenose dolphins (Barratclough *et al.,* 2021, Robeck *et al.,* 2021, Barratclough *et al.,* 2024), humpback whales (Polanowski *et al.,* 2014), beluga whales (Bors *et al.,* 2021), killer and bowhead whales (Parsons *et al.,* 2023)), terrestrial mammals [e.g. bats (Wilkinson *et al.,* 2021], sheep (Sugrue *et al.,* 2021), elephants (Prado *et al.,* 2021), opossum (Horvath *et al.,* 2022), lemurs, rats, baboons, (Horvath *et al.,* 2023)), birds (e.g. chickens (Raddatz *et al.,* 2021)), reptiles (e.g. clawed frogs (Zoller *et al.,* 2023)) and plankton (Hearn *et al.,* 2021). The output of epigenetic clocks, methylation-based estimates of age or epigenetic age, is thought to be indicative of how well an individual’s body systems are functioning. As such, epigenetic age is a proxy for biological age, which can also be estimated using other biomarkers beyond DNAm.

Lastly, it has also been demonstrated that chronic or acute exposure to environmental stressors can leave identifiable signatures in an individual’s global or regional DNA methylation profile—the pattern of methylation across all or some of the CpG sites being assessed. For example, local methylation differences (differential methylation) have been linked to exposure to heavy metals in great tits (Mäkinen *et al.,* 2022), to being born into low-ranking maternal lineages or during droughts in wild baboons (Anderson *et al.,* 2024), to the introduction of an invasive predator in eastern fence lizards (Schrey *et al.,* 2016), to exposure to anthropogenic disturbance in killer whales (Crossman *et al.,* 2021) and to population origin (wild vs. hatchery) in steelhead trout (Nilsson *et al.,* 2021).

## Methods for analysing DNA methylation data

### Pre-processing methods

Epigenetic studies use quantitative methylation data generated from an assay of chemically treated (bisulfite) extracted DNA. This is part of a workflow involving tissue sampling in the field, several stages of laboratory processing and analysis (DNA storage, extraction and assay), and subsequent data processing using computer software and bioinformatic pipelines. The resulting DNAm data are subject to sources of error/variability that accumulate at each step and that need to be understood to make statistically sound inferences. We consider pre-processing to be the steps involved in generating methylation data from extracted DNA that can be quantitatively analysed.

The HorvathMammalMethylChip40, an Infinium Methylation Array (Illumina Inc), is frequently used for mammalian wildlife EWAS on non-primate, non-model organisms. Like other Infinium Methylation Arrays, it detects cytosine methylation of DNA including at CpG islands (regions of the DNA with a high density of CpG sites), genes and enhancers. This array targets parts of the genome where the CpGs and adjacent sequences are highly conserved across mammal species, with high coverage of conserved sites, so that many of the CpGs can be measured in any given mammal species (Arneson *et al.,* 2022). In the first pre-processing step, DNA is treated with bisulfite that leads to conversion of unmethylated cytosine bases to uracil, leaving methylated cytosines unchanged. The assay then uses two site-specific probes to detect these chemically differentiated states at a particular locus, one probe for the methylated state and one for the unmethylated state, each with fluorescent labelling. The level of methylation at a specific locus (across all cells in the sample) is determined using the proportion of methylated signals relative to the total (methylated and unmethylated signals). These proportions are called the “beta values” and are the values analysed in DNAm studies. Standard pre-processing of methylation data from Infinium arrays uses SeSAMe software (Zhou *et al.,* 2018), which requires some user-defined inputs, for example, what detection $P$-value should be used as a cutoff for probe reliability or detection failure. These choices affect the resulting beta values, but are outside the scope of this review. Instead, we focus on methodological choices, ignoring potential errors in beta values.

### Methods for comparing DNAm profiles across groups

Tests of whether exposure to a stressor affects methylation have been primarily conducted as part of EWAS that aim to identify CpG sites that, either individually or in combination, most strongly differentiate between groups, e.g. case–control studies. Broadly, models testing for differences in methylation can be written as $\mathbf{M}=\mathbf{XB}$ where $\mathbf{M}$ is an $n\times p$ matrix of observed DNAm profiles ($n$ samples, $p$ CpG sites; where methylation across all $p$ sites comprises the DNAm profile of sample $n$), $\mathbf{X}$ is an $n\times k$ matrix of covariates ($k$ covariates, one of which is the treatment variable of interest), and $\mathbf{B}$ is a $k\times p$ matrix of regression coefficients. EWAS simplify this into $p$ models of the form ${\mathbf{M}}_i=\mathbf{X}{\mathbf{B}}_i$, where ${\mathbf{M}}_i$ and ${\mathbf{B}}_i$ are the methylation levels and coefficients for CpG site $i$. Approaches can be grouped into four broad categories, depending on whether they identify 1) individual CpG sites or 2) groups of spatially clustered sites known as regions, and whether they do so by testing for differences in 3) mean methylation levels (identifying differentially methylated sites or regions) or 4) the variance of methylation levels (differentially variable sites or regions).

Differentially methylated CpG sites are those with statistically significant differences in mean methylation between groups, whereas differentially variable CpG sites are those with significant differences in the variance of methylation between groups. Each CpG site is considered independent, and differences are identified with standard univariate statistical tests, depending on the study design used and specific properties of the data collected. A difference in mean DNAm might reflect a change in the methylation process, while a difference in the variability of DNAm might reflect the heterogeneity or makeup of the population under study. Common tests for mean differences include $t$-tests, non-parametric alternatives such as the Mann–Whitney or Kolmogorov–Smirnoff test, permutation tests, linear or generalized linear models, and a moderated $t$-test obtained by an empirical Bayes shrinkage of the observed site-specific variances towards a shared prior value (Smyth, 2004). Analogous tests for differences in the variances include Barlett’s, Levene’s, Brown-Forsyth and Breusch-Pagan tests, and an empirical Bayes-based moderated $F$-test. As these are standard statistical tests, we omit details here, but see Teschendorff and Relton (2018). The choice of test depends on features of the data.

Methods that identify differentially methylated or variable regions first quantify the strength of association between each CpG site and the outcome of interest (using the tests above) and then cluster sites identified by the first step together on the basis of spatial proximity. Various approaches exist, but these typically involve (i) defining thresholds such as the minimum genomic distance separating regions, the maximum distance separating sites within the same region, and the minimum size (in terms of differentially methylated sites) of a region and (ii) a spatial clustering algorithm that respects these thresholds. Clustering approaches differ in terms of, for example, whether they spatially smooth over some measure of effect size, either directly using a smoothing function (e.g. *bumphunter* (Jaffe *et al.,* 2012), *DMRcate* (Peters *et al.,* 2015)) or indirectly via autocorrelation functions (*comb-p* (Pedersen *et al.,* 2012)), and whether they hold window sizes fixed at user-specified values or allow these to adapt in response to the data (*Probe Lasso* (Butcher and Beck, 2015), *DMseg* (Wang *et al.,* 2023)).

Three issues that affect these tests are the choice of an appropriate distribution for methylation data, controlling for the large number of statistical tests that are conducted and controlling for potentially confounding covariates.

Distributional choices must take into account that the values typically used as input to statistical analyses are the ratio of methylated to total (methylated and unmethylated, as mentioned in Section 3.1) signal intensity at site $i$, ${b}_i={m}_i/\left({m}_i+{u}_i+a\right)$, where $a\ge 0$ is a regularization constant (used to stabilize beta values when $m$ and $u$ are both small) often set to 100 (Weinhold *et al.,* 2016). Proportional data often exhibit heteroskedasticity, with lower variances closer to the extremes and higher variances around 0.5, which violates the assumptions of many models that assume Gaussian errors. Alternative approaches have been proposed and taken up to some extent, including non-parametric approaches, approaches modelling methylation values as beta or bivariate gamma distributed (Weinhold *et al.,* 2016), and logit transformations of beta values to so-called *M-values*. However, for pragmatic reasons and interpretability, analysis with standard Gaussian methods and beta values remains popular. Differences in results due to choice of response variable are inevitable with so many tests being conducted, but are likely context- and data-dependent (Teschendorff and Relton, 2018; Kruppa *et al.,* 2021).

Adjusting for the large number of independent hypothesis tests conducted by an EWAS is important to control the probability of false positives (type I errors). Typically, this involves adjusting the $P$-value or significance threshold to more conservative levels. Methods for doing so are well-established in statistical theory and not reviewed here (see (Bender and Lange, 2001)). The most popular approach in epigenetic studies is to control the expected proportion of false discoveries (i.e. to $P\le 0.05$), using the approach as in Benjamini and Hochberg (1995).

Potential confounding variables can be included as covariates where appropriate. One potential confounder that has received a lot of attention is the proportion of various cell types making up an epigenetic sample. DNAm profiles are partly determined by the distribution of cell types in a sample, and if these differ systematically, e.g. between cases and controls, then the effect of the control variable on DNAm is confounded with the effect of the control variable on the cell type distribution. Recovering the direct effects of the control variable on DNAm requires controlling for heterogeneity in cell-type distributions (supplementary material Section S1.1 Cell-type deconvolution), as has been done recently in some studies, e.g. (Tomusiak *et al.,* 2024).

### Methods for predicting chronological age from DNAm

First-generation epigenetic clocks (FGECs) predict chronological age (hereafter age) using DNAm as input (Yousefi *et al.,* 2022). These were developed in the human literature, not specifically to predict age (since this is nearly always known) but rather as an intermediate step in assessing aging or health (see next section). Examples are (Horvath, 2013), (Hannum *et al.,* 2013), (Zhang *et al.,* 2019) and (Bernabeu *et al.,* 2023). FGECs have been enthusiastically taken up in the wildlife literature as a less invasive way of estimating animal age, which in many cases is unknown, and to infer health. Age is a basic demographic parameter widely used in population ecology, reproductive studies, and conservation and management, and can sometimes only be estimated by invasive or destructive methods or not at all. A less invasive, cost-effective way to measure animal age is therefore of enormous practical value.

One basic approach for modelling age is to fit a penalized regression model to a cohort of known-age individuals with chronological age as a response and DNAm as a predictor. This model can then be used to predict age in unknown-age individuals. Age may be used as is or transformed, but must be known. The original (Horvath, 2013) clock used a piecewise transformation, imposing a logarithmic dependency until 20 years of age (nominally, adulthood) and a linear dependence thereafter. This transformation continues to be used (Zoller and Horvath, 2024), usually with the age of sexual maturity included as a user-defined threshold separating logarithmic and linear regimes. Other common transformations are the log or square root of age (Bernabeu *et al.,* 2023, Barratclough *et al.,* 2024). These transformations all aim to improve predictive accuracy. Alternatively, age can be expressed in relative terms by dividing raw age by the maximum lifespan of the species, where known, which facilitates comparisons between species.

DNAm is high-dimensional with many strong correlations across CpG sites. An early FGEC (Horvath, 2013) used the elastic net (Zou and Hastie, 2005), a penalized regression model that performs a combination of regularization and feature selection, to produce a clock with fewer CpG sites. This approach has been used by most of the FGECs developed subsequently; other approaches have also been used such as (unpenalized) multiple regression (Polanowski *et al.,* 2014; Weidner *et al.,* 2014), random forests and a hybrid random forest-penalized regression approach (Barratclough *et al.,* 2024), support vector machines (Freire-Aradas *et al.,* 2022), and, increasingly, deep neural networks (Galkin *et al.,* 2021; De Lima Camillo *et al.,* 2022; Prosz *et al.,* 2024).

This basic approach of using penalized regression to predict age from DNAm has now been applied to produce dozens of epigenetic clocks in humans (Horvath, 2013, Hannum *et al.,* 2013, Zhang *et al.,* 2019, Freire-Aradas *et al.,* 2022, Mboning *et al.,* 2024) and for many mammalian and non-mammalian species (see Section 2). Most FGECs select from a suite of methodological extensions that have been shown to improve predictive accuracy, including pre-screening, principal component analysis and hierarchical clustering. Although the elastic net performs its own variable selection, a pre-screening step filtering CpG sites before inclusion into the model has been shown to improve accuracy (Li *et al.,* 2022; Bernabeu *et al.,* 2023). Pre-selection conditions can include: removing CpG sites with modal beta values away from 0 or 1, which can imply technical errors (Zoller and Horvath, 2024); removing CpG sites to mitigate confounders in the study; including only those CpG sites situated in genes involved with chosen functional paths (Ying *et al.,* 2024b) or sites that pass some user-specified threshold on a univariate test of association with age (Dabrowski *et al.,* 2024); or those with high between-replicate reliability as calculated by an intraclass correlation coefficient, where multiple replicates are available (Higgins-Chen *et al.,* 2022). Pre-selection may also be informed by genetic theory; for example (Ndhlovu *et al.,* 2024), filter CpGs that map to retroelements such as human endogenous retroviruses and long interspersed nuclear elements on the human genome whose methylation patterns are subject to age-related drift. Using principal components calculated from CpG-level DNAm data as input, rather than CpG-level DNAm data directly, improved the accuracy of both chronological and epigenetic age predictions (Higgins-Chen *et al.,* 2022). Hierarchical clustering methods have also been used to remove “outlying” samples that appear excessively dissimilar to other samples (Zoller and Horvath, 2024).

Methods applied to produce FGECs for human age prediction have generally been used directly for non-human FGECs with little or no adaptation. FGECs primarily differ according to what kind of samples they are constructed from (e.g. single vs multiple cells, tissue types, see (Simpson and Chandra, 2021)) and also, primarily for human studies, the number and profile of the sampled participants. These in turn influence which CpG sites are selected, how widely the clocks can be expected to generalize, and whether they are sensitive to external factors, primarily cell-type proportions. Many clocks are species- and tissue-specific, while others have been constructed from samples that span multiple tissue types (Horvath, 2013; De Lima Camillo *et al.,* 2022; Prosz *et al.,* 2024) or species (Lu *et al.,* 2023) with a view to more general application. A common practice for animal studies is to construct an FGEC for the species of interest, and another dual-species human–animal FGEC using relative age (age divided by maximum lifespan) as a response and samples from both the species of interest and human tissue to fit the model (Sugrue *et al.,* 2021, Horvath *et al.,* 2023, Zoller and Horvath, 2024). This allows for comparison of aging between species, which can help understand the underlying processes. Tissue-specific FGECs have been developed primarily using samples of blood (Hannum *et al.,* 2013, Weidner *et al.,* 2014) and skin (Parsons *et al.,* 2023, Barratclough *et al.,* 2024) but also brain, liver, ear and tail (Horvath *et al.,* 2022, Sugrue *et al.,* 2021). FGECs may also differ in terms of the methodological choices around pre-filtering, use of principal components, etc., as discussed above.

### Methods for predicting health from DNAm

From the outset, the primary goal of epigenetic clocks in the human literature has been to provide a summary measure of health, rather than to predict chronological age. Nearly, all epigenetic clocks use chronological age as a baseline, but there are several ways in which age can be used, as well as other approaches for developing a baseline. Chronological age is a useful baseline not least because it is an objective value.

#### Age acceleration

Having produced a DNAm-based prediction of age for an individual, its epigenetic age, a natural question is (i) how this compares to the individual’s chronological age, if this is known and (ii) what any differences might imply for the health of the individual; however, health is defined.

Epigenetic clocks have addressed these questions by defining age acceleration as changes in methylation, at sites selected as markers for aging, that are faster than expected on the basis of chronological age. Age acceleration is measured either by the difference (difference-based) between epigenetic age and chronological age (Horvath, 2013), or by the residual from a (usually, but not always, linear) model (model-based) predicting epigenetic age from chronological age and possibly additional control variables e.g. cell-type proportions (Horvath *et al.,* 2014). In either case, the resulting quantity is referred to as an age acceleration residual (AAR). We use the terms difference-based and model-based AARs to distinguish between the two.

Positive AARs are indicative of relatively fast epigenetic aging, negative AARs suggest slower aging and the magnitude of an individual’s AAR reflects the relative speed of their epigenetic aging. If epigenetic aging reflects the status of the underlying tissue and supporting physiological processes, then one may expect some association between AARs and external indicators of health, e.g. mortality or disease risk. Since these markers are correlated with chronological age, which is highly correlated with epigenetic age, most epigenetic clocks seek external validation by demonstrating a significant relationship between AARs and health indicators, after controlling for potentially confounding covariates (e.g. chronological age, body mass index, smoking, prior history of cancer). Many relationships of this form have been established in the human literature (see Horvath and Raj, 2018 and Martínez-Magaña *et al.,* 2024).

Treating residuals from age-predicting models, i.e. FGECs, as *de facto* indicators of health raises the issue of dependency on model accuracy, which is influenced by factors such as sample size, technical noise and statistical methodology, that are unrelated to health. AARs from FGECs have been shown to become smaller and more weakly associated with mortality as the size of the training dataset increases (Zhang *et al.,* 2019). More recently, Dabrowski *et al.* (2024) have shown that this pattern emerges not because of a larger sample size *per se*. Instead, it is because a larger sample size permits the selection of CpG sites that are predictive of factors that affect health, like smoking. These factors improve chronological age prediction for a subset of individuals but do so by reducing the component of the residual that is predictive of health. We explore this issue more fully in Section 5.2.1.

Partially in response to these methodological issues, and partially in pursuit of stronger and more durable associations with health or survival outcomes, a suite of so-called second-generation epigenetic clocks (SGECs) have been proposed. Rather than building predictive models of chronological age, SGECs aim to predict health biomarkers directly from DNAm inputs.

#### Second-generation epigenetic clocks

An obvious question arises with SGECs: if they model health markers directly, in what sense are they still clocks? Answering this question requires mapping relative changes in predicted biomarkers onto the passing of chronological time. As there are various ways to link health markers to DNAm and to standardize units of time, many SGECs are theoretically possible. This section reviews three widely used (in human studies) SGECs to highlight what they have in common and how they differ: *PhenoAge*, *GrimAge* and *Dunedin Pace of Aging*. These methods have been developed to link health conditions and exposure to stressors in humans and would be exciting applications to wildlife settings where health biomarker data are available.

PhenoAge (Levine *et al.,* 2018) consists of three distinct stages. The first stage estimates the parameters of two independent proportional hazards models that predict mortality risk: (i) one model predicts mortality risk from 42 clinical biomarkers capturing phenotypic variability (e.g. metabolic, renal, immune function) as well as chronological age (variable selection retains 9 biomarkers and age) and (ii) the other model uses only chronological age to predict mortality risk. The second stage creates a standardized unit of phenotypic age by equating the 10-year mortality risk derived from the two models fitted in the first stage. In this step, age in the age-only model is left as an unknown parameter, while all other parameters are known. Solving this equality provides the chronological age at which the predicted mortality risk from age alone is equal to the predicted mortality risk from all biomarkers as well as age. This is termed an individual’s PhenoAge. Individuals whose PhenoAge exceeds their chronological age are at a higher risk of mortality than would be expected from their age alone. Note that no DNAm data have been used to this point. The third stage fits an elastic net model with PhenoAge as a response and DNAm as a predictor. Predictions of PhenoAge from this model are DNA-based and are referred to as DNAm-PhenoAge. AARs can be calculated from either PhenoAge or DNAm-PhenoAge in the usual way.

GrimAge (Lu *et al.,* 2019) also consists of three stages. The first stage fits 89 independent models, each of which predicts a biomarker (88 plasma proteins and smoking pack-years) from DNAm, age and sex. Biomarkers with sufficiently high correlations between predicted and observed values in a test set ($\ge$0.35) are retained in the next stage (12 protein biomarkers and smoking pack-years). The second stage fits a proportional hazards model that predicts mortality risk from DNAm-based predictions of the biomarkers from the first stage, as well as age and sex. In the third stage, predictions of mortality risk are transformed into estimates of epigenetic age by normalizing the linear predictor from the second-stage hazard model, which provides an index of mortality risk (the log relative risk compared to a hypothetical individual whose linear predictor is zero), having the same mean and variance as that of chronological ages in a training dataset. Values of the linear predictor following this transformation are referred to as *DNAm-GrimAge*. Again AARs can be calculated in the usual way.

Dunedin Pace of Aging (Belsky *et al.,* 2020; Belsky *et al.,* 2022) (DPoA) is a three-stage model mainly defined by its use of a longitudinal dataset that tracks various standardized health measures (18 blood-chemistry and organ-system biomarkers) at age 26, 32 and 38 (Belsky *et al.,* 2015; Belsky *et al.,* 2020), later augmented by DNAm data at age 45 (Belsky *et al.,* 2022). The first stage fits 18 independent linear mixed effects models, each of which predicts a biomarker from age. Due to the longitudinal nature of the input data, age enters the model both as a fixed effect (the average effect of being a certain age on the response) and a random slope (the individual-specific effect of aging one year on the response biomarker). Larger positive values of the random slope indicate an individual whose biomarkers have changed more rapidly. To convert this into a measure of the rate of aging, the second stage defines the sum of the random slopes across all 18 biomarkers as the pace of aging (PoA), and scales these to have a mean of one, with no standardization of the variance. This implies that the pace of (biological) aging matches the pace of chronological aging on average across the cohort. The third stage fits an elastic net model with PoA as a response and DNAm as a predictor. Predictions of PoA from this model are DNA-based and are referred to as PoAm. AARs can be calculated from either PoA or PoAm in the usual way.

The three SGCEs all perform three core tasks: biomarkers are related to DNAm; a temporal rate aspect is introduced (meaning a ratio in which the denominator is some unit of chronological time); and this rate is normalized to establish the baseline for a unit of epigenetic time. How they do this, and in what order, differs substantially between the methods. GrimAge relates biomarkers to DNAm directly, whereas in PhenoAge and DPoA biomarkers are used to establish a phenotypic measure of aging, which is then regressed on DNAm. PhenoAge and DPoA therefore provide both non-DNAm and DNAm-based measures of biological aging, while GrimAge provides only the latter. GrimAge and PhenoAge use DNAm to predict a response (time-to-death) that occurs after the collection of DNAm data, while DPoA uses the rate of change in biomarkers over the 12 (DPoA, (Belsky *et al.,* 2020)) or 20 (DPACE, (Belsky *et al.,* 2022)) years prior to collection of DNAm. Thus, DPoA provides a measure of retrospective aging, while GrimAge and PhenoAge are, in some sense, forward-looking. Normalization of epigenetic aging in PhenoAge is made possible by fitting age-only and age-and-biomarker models; in GrimAge and DPoA, it is achieved by scaling with summary statistics from a training dataset. Interpretations of relative changes differ. A higher PhenoAge means a higher predicted 10-year mortality risk. A higher GrimAge means a higher log relative risk of all-cause mortality. A higher DPoA means a higher rate of change in selected biomarkers over the previous 12 (DPoA, Belsky *et al.,* 2020) or 20 (DPACE, Belsky *et al.,* 2022) years.

The combinations of modelling choices above are by no means the only possible ones, nor is any one approach obviously better than the others. They are collections of choices made with the primary goal of achieving stronger correlations with external health outcomes than those achieved using FGECs.

#### Other clocks

Here, we briefly review two recent clocks that point towards promising directions in epigenetic research in both human and wildlife settings: the development of mechanistic models of methylation dynamics (*ProbAge*) (Dabrowski *et al.,* 2024) and using causal inference to disentangle the bi-directional relationship between methylation and biological age (Ying *et al.,* 2024a). These two new approaches are exciting because they make it possible to make inferences about the mechanisms underlying changes in methylation profiles, and overcome the issue of confounding between aging due to passive drift and stressor-driven changes.

Mechanistic clocks attempt to capture the essential aspects of the process of methylation while remaining tractable and clinically useful. The core of the approach in (Dabrowski *et al.,* 2024) (referred to as ProbAge) is to model transitions between two cell states, methylated and unmethylated, using a continuous-time Markov model. Under this model the proportion of methylated cells at a CpG site $i$ for an individual $j$ measured at time ${t}_j$ (age), denoted by ${m}_{ij}\left({t}_j\right)$, follows a beta distribution $B\left({m}_{ij}\left({t}_j\right);{\mu}_i\left({t}_j\right),{\sigma}_i\left({t}_j\right)\right)$. The likelihood of observing a particular set of DNAm values at one site across all $J$ individuals, denoted ${\mathbf{m}}_{\mathbf{i}}=\left({m}_{i1}\left({t}_1\right),\dots, {m}_{iJ}\left({t}_J\right)\right)$, is given by $L\left({\mathbf{m}}_{\mathbf{i}}\right)={\prod}_{j=1}^JB\left({m}_{ij}\left({t}_j\right);{\mu}_i\left({t}_j\right),{\sigma}_i\left({t}_j\right)\right)$. Closed-form expressions allow for the estimation of ${\mu}_i\left({t}_j\right)$ and ${\sigma}_i\left({t}_j\right)$ in terms of five parameters: ${\eta}_i$, the proportion of methylated cells as $t\to \infty$ (steady state); ${p}_i$, the proportion of methylated cells at $t=0$ (initial methylation proportion); ${\omega}_i$, the rate of methylation state transition; ${N}_i$, the total number of cells; and ${c}_i$, a constant term. We collect these parameters into the vector ${\theta}_i$.The approach consists of two stages that model change in methylation at the site level and the individual level, respectively. The first stage uses DNAm data at each site, ${\mathbf{m}}_{\mathbf{i}}$, to independently estimate the parameters associated with that site, ${\theta}_i$, by maximum likelihood. Note that at this stage the same parameter estimates apply to all individuals, a clearly unrealistic condition but one that forms the basis for the second stage. The second stage considers two types of changes potentially affecting an individual’s methylation dynamics. The first is a differential transition rate between methylated and unmethylated states, expressed by ${\alpha}_j{\omega}_i$ where ${\alpha}_j$, termed the acceleration parameter, is interpreted as the proportional increase in speed of methylation transitions. $\alpha$ is an acceleration parameter in that it captures how fast an individual’s epigenome is changing, relative to the rest of the population (constant over all CpG sites $i$), and can be though of as an intercept-type term in a linear regression model. The second is a shift in the initial and steady state methylation proportions, expressed as ${\eta}_i+{\beta}_j$ and ${p}_i+{\beta}_j$ respectively. $\beta$ is referred to as the bias parameter since it captures global changes in methylation levels and can be thought of as a slope-type term in a linear regression model. Note that values of ${\alpha}_j$ and ${\beta}_j$ can differ between individuals, i.e. they allow for individual-specific methylation dynamics, but within any individual values are constant over all CpG sites. Assuming independence between individuals, parameter values for ${\alpha}_j$ and ${\beta}_j$ can again be estimated by maximum likelihood.

This clock shows associations with age-related health outcomes that are comparable to SGECs, but uses the same input data as FGECs, i.e. without biomarkers. The ability to separately estimate the acceleration and bias components of methylation is a major advantage because it makes it possible to distinguish between aging and stressor-induced shifts in methylation. This confounding is an inherent limitation in FGECs: combinations of negative bias (global decrease in DNAm) and increased acceleration (observed, e.g. for smoking) or positive bias (global increase in DNAm) and decreased acceleration (observed, e.g. for total cholesterol) cancel each other out to some extent masking any effects that are present. As a consequence, methods that cannot decouple acceleration and bias tend to find no or weak associations between methylation and epigenetic age. Furthermore, ProbAge provides a flexible framework that can be extended, e.g. to allow for acceleration and bias to depend on covariates. Disadvantages include that the model needs to be fit to each new dataset, i.e. it does not provide a fitted model that can be used to predict with a new dataset of DNAm profiles, and it does not provide an estimate of epigenetic age.

Statistical (or machine learning) techniques that model associations between DNAm and age or health face the challenge that the direction of causality in these relationships can go both ways (Teschendorff and Relton, 2018). Causal inference provides a set of tools to potentially isolate the directionality of effects. (Ying *et al.,* 2024a) use Mendelian Randomization, which exploits randomness arising in genetic variants to effectively run a randomized controlled trial from which causal inferences may be drawn (Sanderson *et al.,* 2022). CpG sites that are causally related to eight age-related traits are identified, with very little overlap between these and CpG sites selected by other FGECs and SGECs. An FGEC-like elastic net is then used to predict chronological age from DNAm, except that the standard elastic net penalty term is augmented to include causal effect sizes, with a user-specified parameter determining the weight assigned to these effects. Note that causal effect sizes could also be used as a pre-selection condition, and augmenting the elastic net penalty is a general strategy that can be used with other measures used to pre-select CpG sites.

#### Direct prediction of phenotypes from DNAm

DNAm-based trait score models (Nabais *et al.,* 2023) predict a phenotype of interest from DNAm, in much the same way that FGECs predict age. Indeed, if predicting a particular phenotype or health outcome is the primary goal, then there is no real need for estimating epigenetic age and directly modelling this as a function of DNAm inputs is the more natural approach. (Nabais *et al.* (2023) provide a review of phenotypes that have been predicted from DNAm in humans: these include body mass index (BMI) (McCartney *et al.,* 2018), smoking (Bollepalli *et al.,* 2019), Alzheimer’s (Smith *et al.,* 2021) and cognitive ability (McCartney *et al.,* 2022).

The broad approach used to construct trait score models is similar to those used to construct FGECs, with penalized regression models widely used. As age is no longer the response, models often control for age, together with sex. Some models attempt to isolate epigenetic effects by also controlling for genetic effects, either by including genetic predictors as control variables (McCartney *et al.,* 2018) or with mixed effects models that account for genetic relationships (Trejo Baños *et al.,* 2020; Vallerga *et al.,* 2020). These models have typically been used to predict a single phenotype, and thus do not provide a single summary metric of health, such as age acceleration. However, various health indicators could be condensed into a single health metric (Schwacke *et al.,* 2024) and used as a response variable.

## Case study: age and health prediction from wildlife DNAm

In this section, we illustrate some of the methods described so far by applying them to an existing wildlife dataset from bottlenose dolphins (*Tursiops* spp.). We compare the model output, discuss model assessment and highlight some of the analytical limitations and opportunities that arise. The dataset comes from Barratclough *et al.* (2024), who carried out an analysis focused on age estimation and relating age acceleration and health. We expand on that analysis here, with additional FGECs, SGECs and trait score models.

### Data

We use the methylation data reported in Barratclough *et al.* (2024), collection and processing steps of which we summarize in the supplementary material for completeness (S1.2 Methods for sample collection and processing). The data originate from 476 skin samples collected from 439 individual bottlenose dolphins. Of these, 426 came from 389 free-ranging animals from eight management stocks (four of Tamanend’s bottlenose dolphin, four of common bottlenose dolphin) sampled as part of catch-and-release health assessment or remote biopsy studies), while 50 samples are from 50 individuals at the US Navy Marine Mammal Program.

Chronological age was known for 40 of 50 Navy dolphins born at the facility and for 87 of 429 free-ranging animals that were observed soon after birth. Ages for other dolphins were estimated from dentinal analysis of growth layer groups ($n=280$) (Hohn *et al.,* 1989) or from dental radiography $\left(n=59\right)$ (Herrman *et al.,* 2020). (Barratclough *et al.,* 2024) defined a quantitative measure of uncertainty for each age estimation technique (ranging from 0 to 1) that we used in some settings to downweight age estimates with higher uncertainty. Depending on the technique used, the weighting factor was based on a combination of estimated age, time since age was estimated, and (for growth layer groups) mean square error of age estimate.

In addition to the methylation data, health data were also collected for the cohort of free-ranging animals during the same catch-and-release studies used to collect skin samples. To test for associations between age acceleration and health, we used the Veterinary Expert System for Outcome Prediction (VESOP) score (Schwacke *et al.,* 2024), which aggregates a range of biomarkers into a score between 0 and 1 representing predicted one-year survival probability, as well as a subset of markers used as inputs to the VESOP score. These include markers of inflammation (neutrophil count, alkaline phosphatase), metabolism (cholesterol), neuroendocrine status (cortisol) and lung disease (scored from ultrasound).

### Methods

The analysis was conducted within a 10-fold cross-validation framework, where each dolphin was allocated using a balanced-by-site (where site is population) random design (see below) to one of ten subsets of approximately equal size, called folds. The dolphins in each fold were used as test data. The dolphins from the nine remaining folds were used for training and validation (hyperparameter selection), with 85% of the dolphins randomly allocated for training and the remaining 15% used for validation (supplementary material S1.4.1). This resulted in ten training datasets, each associated with its own validation and test dataset. Each dolphin appears in exactly one test dataset, and nine times across training and validation datasets. For dolphins sampled multiple times, all observations were allocated to the same fold. Validation datasets were used to select model hyperparameters, higher-level parameters specifying the configuration or architecture of a particular model. Test datasets were used to evaluate the expected model performance on unseen data. Folds were constructed to preserve the proportion of dolphins from populations without known acute stressor exposure events, whose populations are assumed to be comparatively healthy (US Navy dolphins; Charleston, South Carolina; Sarasota, Florida; Indian River Lagoon, Florida, following (Barratclough *et al.,* 2024)), and referred to hereafter as control populations, within each fold. The remaining populations (St. Joseph Bay, Florida; Mississippi Sound, Mississippi; Barataria Bay, Louisiana; Southern Georgia) had been exposed to stressor events, with known effects on health (Barratclough *et al.,* 2024; Schwacke *et al.,* 2024), referred to hereafter as exposed populations.

Within this framework, sensitivity of results to various modelling choices was assessed. The choices relate to transformations of the dependent variable, definition of the training sample, and accounting for uncertainty in observed chronological ages. The dependent variable, age, was either modelled directly or transformed using the logarithm of age before somatic maturity [assumed to be 15, (Barratclough *et al.,* 2024)] and linearly thereafter (supplementary material S1.4.1). Training was either over all individuals, or restricted to individuals from control populations, or a random sample the same size as the control sample, but drawn from both control and exposed populations (to disentangle the effects of sample size from the effects of including exposed individuals). The model was trained either with or without sample weights inversely related to the uncertainty of age estimates (Barratclough *et al.,* 2024). Results are reported for each of these 12 modelling conditions (with or without age transformation, three training samples, with or without sample weights).

Hyperparameters for each model were chosen by grid search, i.e. by fitting independent models for all hyperparameter combinations and choosing the combination that minimized median absolute error across the validation datasets (supplementary material S1.4.1). For all models, the number of pre-filtered methylation sites passed to the model was a hyperparameter whose value was selected from a set of candidate values (500, 1000, 2000, 31 139). Pre-filtered sites were selected on the basis of absolute correlations with the outcome of interest (age or health), calculated from the training data. Elastic net and random forest models have additional hyperparameters discussed below.

#### First-generation clocks

Predictions of chronological age based on methylation data were generated using both elastic net regression and random forest models. Elastic net regularization was applied using a mixing parameter $\alpha$ that controls the balance between LASSO and ridge regression penalties, either set to a default value of $\alpha =0.5$ or optimized using the cross-validation procedure described above with a candidate set $\alpha \in \left\{\mathrm{0.01,0.1,0.2},\dots, \mathrm{0.9,0.99}\right\}$. The optimal $\lambda$ parameter for regularization was determined via cross-validation within the training set. Random forest models were trained using 1000 trees. The proportion of available features randomly selected at each split using a hyperparameter set either to one-third of the total number of available features, or chosen by cross-validation over the candidate set $\left\{1/6,1/3,1/2,2/3\right\}$.

#### ProbAge

Parameters of the ProbAge model (see section 3.4.3 and supplementary material S1.4.2) were estimated by (Dabrowski *et al.,* 2024) using Bayesian maximum *a posteriori* (MAP) estimation, equivalent to maximizing the product of the likelihood (e.g. of observing the methylation levels in an individual across sites) and a prior distribution. As software used to implement ProbAge did not accommodate sample weights, we wrote our own maximum likelihood implementation that directly maximized the ProbAge likelihoods using the Nelder–Mead algorithm in R function *optim*. To avoid having to constrain parameters, log and logit transformations were applied to all non-negative parameters and unit-interval parameters. Note that in ProbAge, age enters the model via an exponential term, so non-linear aging dynamics are incorporated by default. Applying a log transform to age therefore converts this back to a linear dependency—unlike conventional clocks, where log transformations are used to introduce non-linearity.

#### Existing clocks

We included epigenetic age predictions from three existing clocks using the R package *MammalMethylClock* (Zoller and Horvath, 2024). These were (in order of increasing generality) the bottlenose dolphin clock by (Robeck *et al.,* 2021) (constructed from an independent set of samples to ours), a cetacean clock constructed from 13 species, including bottlenose dolphins, by (Zoller *et al.,* 2025), and the pan-mammalian clock of (Lu *et al.,* 2023).

#### Ensemble models

Ensemble predictions, created by aggregating predictions across several models, are widely used in predictive modelling. To test their efficacy here, we created two ensemble models from the output of the three existing clocks and optimized elastic net and random forest models. One model used the unweighted mean of the five predictions produced by these models. The other “optimized” model used a weighted mean with weights obtained from the coefficients of a linear regression of chronological age on the training-set predicted ages of each of the five clocks making up the ensemble. Weights were calculated separately within each combination of modelling choices described above.

#### Assessment of epigenetic clocks

Clocks were assessed on their ability to predict chronological age, and their associations with health indicators after controlling for age and sex.

Ability to predict chronological age was assessed using the correlation between chronological and epigenetic age, the median relative bias and median absolute value of AARs across observations for each clock. AARs were calculated in three ways: (i) using the observed difference between epigenetic and chronological age for each sample; (ii) using the residual from a linear regression of chronological age on out-of-sample epigenetic age predictions, i.e. those obtained from the test fold and (iii) using residuals from a generalized additive model (GAM) fitted to the same relationship as in (ii). GAMs were implemented in R package *m*gcv and used thin plate regression splines with basis dimension $k=4$. In all cases, AARs were orientated so that positive values indicate higher-than-expected epigenetic age, i.e. as epigenetic age minus chronological or predicted mean epigenetic age. For ProbAge, which does not produce a prediction of epigenetic age, only the correlation between chronological age and each of the ProbAge outputs (age acceleration and bias) are reported.

Associations between AARs and health were investigated for illustrative purposes using a subset of the health markers used by (Schwacke *et al.,* 2024): VESOP health scores (an aggregate health metric representing predicted one year survival probability); and binary indicators of lung disease (scored from ultrasound), endocrine dysfunction (cortisol values below the 5th percentile), anaemia, high globulin, low glucose and neutrophilia (see Table 1 in (Schwacke *et al.,* 2024) for details). For each combination of health outcome and type of AAR, we first isolated the part of the health outcome that could not be explained by chronological age and sex by taking the residuals of a logistic regression model in which the health outcome was the response and age and sex were predictors. Again, residuals were oriented so that positive residuals were indicative of worse-than-expected health. We then calculated the correlation between these residuals and each AAR. $P$-values indicating statistical significance of correlations were corrected using the false discovery rate method (Benjamini and Hochberg, 1995). For ProbAge, we used the age acceleration and bias parameters directly as AARs, i.e. without fitting any of the models in (i) to (iii) above.

**Table 1:** MAE in years (lower quartile, upper quartile) and relative bias (proportional error) of DNAm age stratified by age class from existing and modelled first-generation clocks

	(a) Median absolute error				
Clock	Calf($n=28$)	Subadult($n=273$)	Adult($n=110$)	Older($n=65$)	All($n=476$)
BND	3.7 (3.3; 4.4)	1.8 (1; 2.9)	3.6 (1.9; 5.2)	10.7 (6.4; 15.1)	2.6 (1.4; 4.9)
Cetaceans	3.2 (2.8; 4.2)	2 (1; 3.7)	2.7 (1.2; 3.9)	5.1 (2.6; 9)	2.5 (1.2; 4.3)
Mammals	1.5 (0.9; 2)	1.7 (0.7; 3.2)	2.8 (1.1; 5)	9.8 (6.8; 15.5)	2.2 (1; 4.7)
Random forest	0.5 (0.2; 0.7)	1.6 (0.6; 3.3)	2.3 (1.4; 4.3)	7.1 (4.3; 12.2)	2 (0.8; 4.5)
Elastic net	0.2 (0.1; 0.7)	1.3 (0.6; 2.9)	3.4 (1.6; 5.2)	4.5 (2.2; 8.3)	1.7 (0.7; 4)
Random forest opt	0.3 (0.1; 0.6)	1.5 (0.6; 3)	2.4 (1.2; 4.7)	6.3 (3.4; 10.1)	1.9 (0.7; 4.2)
Elastic net opt	0.2 (0.1; 0.5)	1.2 (0.5; 2.8)	3.4 (1.6; 6.5)	3.9 (1.5; 9.6)	1.6 (0.6; 4.1)
Ensemble	1.7 (1.5; 2.1)	1.4 (0.7; 2.5)	2.1 (0.9; 3.7)	6.8 (3.6; 10.8)	1.8 (0.9; 3.5)
Ensemble opt	0.4 (0.3; 0.8)	1.9 (0.8; 3.6)	2.9 (1.5; 5.6)	5.6 (3.3; 9)	2.3 (0.9; 4.9)
	(b) Median % relative bias				
Clock	Calf	Subadult	Adult	Older	All
BND	220 (183; 610)	17 (−6; 54)	−16 (−25; −7)	−32 (−40; −22)	−1 (−20; 39)
Cetaceans	203 (151; 581)	27 (6; 62)	3 (−10; 15)	−13 (−23; −1)	15 (−4; 51)
Mammals	116 (65; 351)	18 (−2; 51)	−7 (−21; 4)	−32 (−44; −24)	4 (−16; 36)
Random forest	39 (24; 118)	14 (−3; 43)	4 (−12; 13)	−23 (−31; −14)	5 (−14; 30)
Elastic net	17 (−3; 50)	4 (−13; 27)	2 (−15; 18)	−10 (−22; 1)	2 (−15; 21)
Random forest opt	27 (5; 99)	12 (−5; 36)	3 (−11; 15)	−16 (−28; −10)	5 (−13; 28)
Elastic net opt	11 (−15; 30)	−3 (−18; 18)	0 (−17; 20)	−6 (−24; 2)	−2 (−18; 17)
Ensemble	110 (88; 343)	16 (0; 39)	−3 (−15; 8)	−20 (−29; −13)	7 (−11; 33)
Ensemble opt	3 (−119; 18)	15 (−9; 42)	7 (−9; 20)	−16 (−26; −8)	7 (−16; 31)

Age classes are defined as $\le$2 calf ($n=28$); 2 $<$ subadult $\le$15 ($n=273$); 15 $<$ adult $\le$25 ($n=110$); older $>$ 25 yrs ($n=65$). Results are for models trained on control individuals using log-linearly transformed age and sample weights

#### Trait score models

Trait score models directly modelling health indicators as a function of DNAm were implemented using the same elastic net regression and random forest models described in Section 4.2.1, substituting health for age as the dependent variable. Seven binary health outcomes were used as responses: anaemia, low cortisol, high globulin, low glucose, lung disease, neutrophilia and a VESOP score below the 20th percentile (corresponding to one-year predicted survival $<$ 0.925). All of these outcomes were relatively rare (less than 20% of the data). Discretizing VESOP scores and health outcomes was not essential but simplified presentation of the results.

Elastic net logistic regression models and random forest classifiers were independently fit to each binary outcome, with model hyperparameters (elastic net mixing parameter $\alpha$, random forest split proportion ${m}_{\mathrm{try}}$) again either set to default values ($\alpha =0.5$, ${m}_{\mathrm{try}}=1/3$) or optimized over the same candidate sets described previously ($\alpha \in \left\{\mathrm{0.01,0.1,0.2},\dots, \mathrm{0.9,0.99}\right\}$, ${m}_{try}\in \left\{1/6,1/3,1/2,2/3\right\}$). Models were evaluated based on test set accuracy (proportion of all predictions that were correct), sensitivity (i.e. recall: number of true positives divided by positive examples), specificity (number of true negatives divided by negative examples), precision (number of true positives divided by true positives and false positives), F1 score (harmonic mean of precision and recall, ranging between 0 and 100), receiver operating characteristic (ROC) curves and the area under these curves (AUC). Optimal thresholds for converting predicted probabilities into binary classifications of health were chosen so as to maximize the F1 score in the validation dataset.

### Results

#### Accuracy of chronological age predictions from epigenetic clocks

Having fit a range of models to the case study data, we can use the predictions to review the accuracy of the different methods. We present overall accuracy for all age classes combined, but also by age class because the accuracy for different age classes can vary substantially. Age predictions from epigenetic clocks we ran were on average within 2–3 years of chronological age for all existing and first-generation clocks (Table 1a), with the elastic net clock producing marginally more accurate predictions. (Barratclough *et al.,* 2024) also present results from fitting a hybrid elastic net-random forest model on this dataset [(Barratclough *et al.,* 2024) Table 2]. All clocks systematically underestimated the age of older individuals and overestimated the ages of calves (Table 1b). Optimization of hyperparameters slightly improved the accuracy of elastic net and random forest clocks. Ensemble predictions performed no more accurately than predictions made by individual clocks.

**Figure 1:**
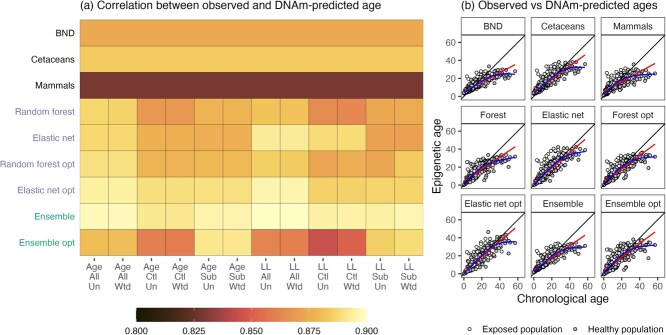
Associations between chronological and epigenetic age across different modelling choices (Age/LL = untransformed/log-linearly transformed age; All/Ctl/Sub = models trained on all individuals/control individuals/subset of all individuals; Un/Wtd = sample weights not used/used). Panel (**a**) shows correlations between clocks and chronological age. First-generation clocks show high correlations (>0.83) across all conditions. Panel (**b**) shows chronological age versus epigenetic age for selected clocks. Solid black, red and blue lines denote the line $y=x$ and curves from fitted linear and generalized additive models respectively.

The strength of association between chronological and epigenetic age showed very little variation over modelling conditions for FGECs (Fig. 1a). Correlations for the optimized elastic net model were between 0.89 and 0.90, while those for the optimized random forest model were between 0.87 and 0.89. Hyperparameter optimization, log-linear transformations of age and accounting for uncertainty in observed ages through sample weights had very little impact on correlations in first-generation clocks. Of existing clocks, predictions from the cetacean clock of (Zoller *et al.,* 2025) and bottlenose dolphin clock of (Robeck *et al.,* 2021) were equally correlated with age (0.88 and 0.87, respectively), with the pan-mammalian clock (Lu *et al.,* 2023) less but still highly correlated (0.83).

**Figure 2:**
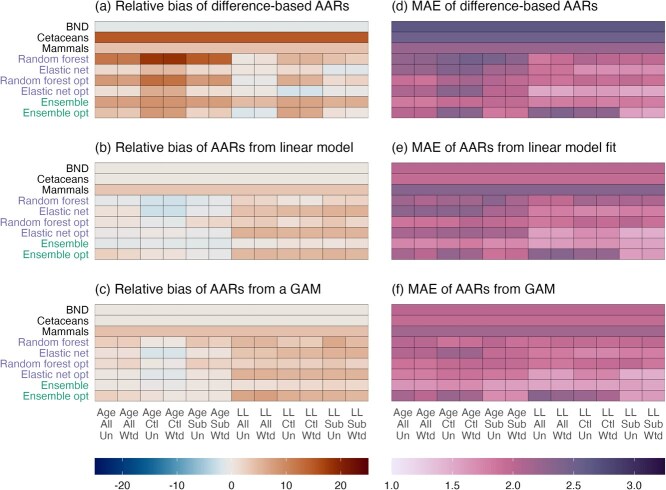
Accuracy of age predictions from epigenetic clocks (vertical axis) across different modelling choices (horizontal axis), as measured by median proportional error (relative bias, the ratio of AARs to chronological age, panels **a**-**c**) and MAE (panels **d**-**f**). Panels (**a,d**) show results for AARs calculated as the differences between observed and predicted age. Panels (**b,e**) and (**c,f**) show results when AARs are model residuals from fitted linear and generalized additive models, respectively. Model conditions: Age/LL = untransformed/log-linearly transformed age; All/Ctl/Sub = models trained on all individuals/control individuals/subset of all individuals; Un/Wtd = sample weights not used/used).

In contrast to the strength of association between chronological and epigenetic age, the median relative bias and absolute error were more sensitive to modelling conditions (Fig. 2a and d). Positive bias (overestimation of age) and absolute errors were larger for models trained on control populations only, and smaller if log-linear age was used as a response. Differences were more pronounced for random forest clocks than those built using the elastic net, and for unoptimized models rather than those with optimized hyperparameters. Bias was largely eliminated by fitting either linear or generalized additive models (Fig. 2b and c). This also had the effect of reducing median absolute errors considerably (Fig. 2e and f).

**Figure 3:**
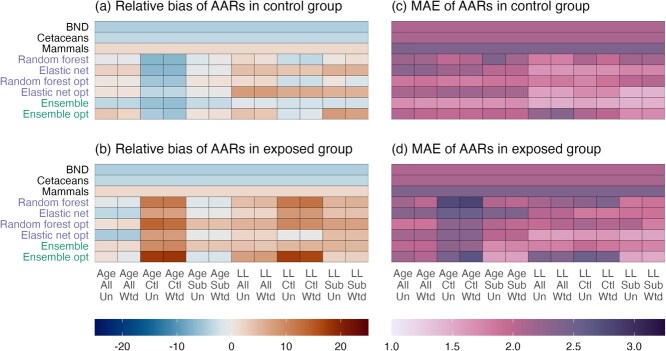
Differences in the accuracy (relative bias, MAE) of age predictions from epigenetic clocks (vertical axis) across different modelling choices (horizontal axis) across different cohorts of the test sample. Panels (**a** and **c**) show accuracy for individuals from control populations. Panels (**b** and **d**) show accuracy for individuals from exposed populations. Model conditions: Age/LL = untransformed/log-linearly transformed age; All/Ctl/Sub = models trained on all individuals/control individuals/subset of all individuals; Un/Wtd = sample weights not used/used).

The larger errors (MAE) observed for models trained on control populations only reflect much less accurate age predictions among individuals from exposed populations, i.e. from outside the training sample (Fig. 3d). Ages of these individuals tended to be substantially overestimated (Fig. 3b). In contrast, errors in the predicted ages of individuals in control populations were roughly equal regardless of how the training sample was composed, particularly for optimized models (Fig. 3c).

#### Associations between age acceleration residuals and health

**Figure 4:**
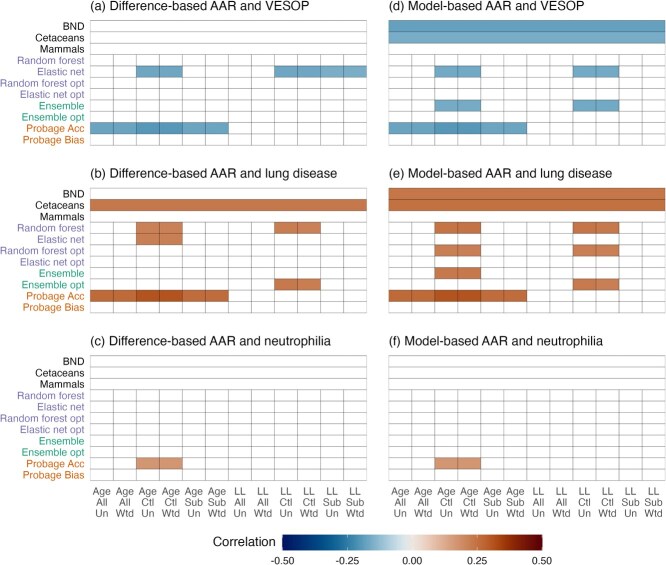
Associations between three health outcomes and age acceleration residuals (AARs) from epigenetic clocks (across different modelling choices (Age/LL = untransformed/log-linearly transformed age; All/Ctl/Sub = models trained on all individuals/control individuals/subset of all individuals; Un/Wtd = sample weights not used/used). Health outcomes are an aggregate health score (VESOP, panels **a** and **d**), an indicator of lung disease (**b** and **e**) and an indicator of neutrophilia (**c** and **f**). AARs are calculated as the difference between epigenetic and chronological age (panels **a–c**) or the residual from a fitted linear model (**d–f**). Results for GAM-based AARs were very similar to those obtained with a linear model and not shown here. Correlations not significant at *P* < 0.05 after adjustments for multiple comparison are whited out.

**Figure 5:**
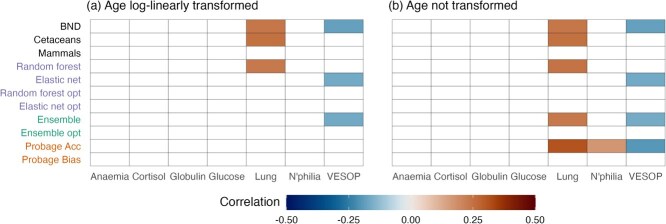
Associations between an expanded set of health outcomes (horizontal axis) and age acceleration from epigenetic clocks (vertical axis) fitted to individuals from control populations using (**a**) log-linearly transformed age and (**b**) untransformed age. Age acceleration is from the residual from a fitted linear model for all clocks except ProbAge, which calculates age acceleration and bias directly. Results are without the use of sample weights. Correlations not significant at *p* < 0.05 after adjustments for multiple comparison are whited out. Note that small differences in lung outcomes shown in Fig. 4 are due to adjusting for different numbers of tests.

Associations between age acceleration residuals and health were moderate and depended both on the epigenetic clocks used and on modelling conditions (Fig. 4). The strongest and most consistent associations were those involving the ProbAge acceleration parameter. Dolphins with higher ProbAge acceleration were more likely to have lung disease and lower VESOP scores ($0.26<\rho <0.32$ and $-0.21<\rho <-0.16$ respectively, using untransformed age). ProbAge was also the only clock associated with any other outcome (neutrophilia, Fig. 5).

Difference-based and model-based AARs from elastic net and random forest clocks showed some significant associations with lung disease and VESOP scores, but only under some model conditions, most commonly training on individuals from control populations (Fig. 4a,b,d,e). AARs from existing clocks were also associated with both lung disease and VESOP scores. The most consistent associations were observed in the case of the cetacean clock of (Zoller *et al.,* 2025), with AARs from the bottlenose dolphin clock of (Robeck *et al.,* 2021) associated with both outcomes only if model-based AARs were used. AARs from the pan-mammalian clock of (Lu *et al.,* 2023) were more weakly associated with health, not achieving statistical significance under any of the conditions (Fig. 4e).

#### Trait score models

**Figure 6:**
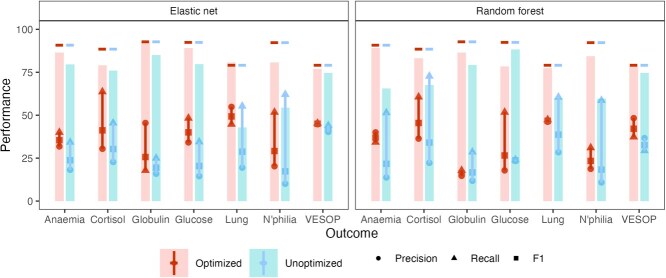
Assessment of model performance in trait score models predicting the presence of seven health outcomes (horizontal axis). Solid vertical lines connect precision (circles) and recall markers (triangles); F1 score (squares) is the harmonic mean of the two. Bars show model accuracy, the proportion of correct classifications, with baseline accuracy obtained by classifying all observations into the majority class shown by horizontal lines. All results show test set (out-of-sample) performance using models with (first bar in each pair) or without (second bar in each pair) hyperparameter optimization on a validation set.

Elastic net and random forest classifiers showed broadly comparable performance across the seven binary health outcomes (Fig. 6). Optimized elastic net models achieved F1 scores ranging from 29 (neutrophilia) to 49 (lung disease), with a mean F1 of 38 across all seven outcomes. In comparison, optimized random forests produced F1 scores between 16 (globulin) and 47 (lung disease), with a mean F1 of 34. Model accuracy was typically lower than the baseline accuracy obtained by classifying all observations into the majority class, reflecting our decision to optimize classification thresholds for the F1 score, which prioritizes a balance between recall and precision (higher value means better model performance). All models were characterized by low precision ($<50$% positive cases correctly identified, except for lung disease), reflecting the difficulty of diagnosing positive cases in highly imbalanced datasets (large differences in the number of cases and controls).

Lung disease, low cortisol and low VESOP scores were the best predicted outcomes for optimized versions of both elastic net and random forest models. Elastic net outcomes were characterized by a balance between precision and recall driving higher F1 scores. In contrast, outcomes modelled with unoptimized models exhibited larger discrepancies between precision and recall, reflecting a tendency of the classifier to overpredict the majority (absence) class. Patterns were more mixed for random forest models. Two of the three best predicted outcomes (lung disease and low cortisol) had large differences between precision and recall, and optimized models were not more balanced than unoptimized ones.

Models trained with optimized hyperparameters provided small but consistent improvements in performance over those using default settings, with greater improvements for elastic net models (mean $\varDelta$F1 across outcomes = 12.0 vs. 7.4 for random forest models). For elastic net models, the largest improvements were observed for lung disease ($\varDelta$F1 = 21; 71%), glucose ($\varDelta$F1 = 20; 96%) and neutrophilia ($\varDelta$F1 = 12; 68%). For random forest models, anaemia ($\varDelta$F1 = 15; 70%) and cortisol ($\varDelta$F1 = 11; 34%) showed the greatest improvements. Improvements were typically largest for outcomes with lower prevalence and driven by improvements in both precision and recall.

**Figure 7:**
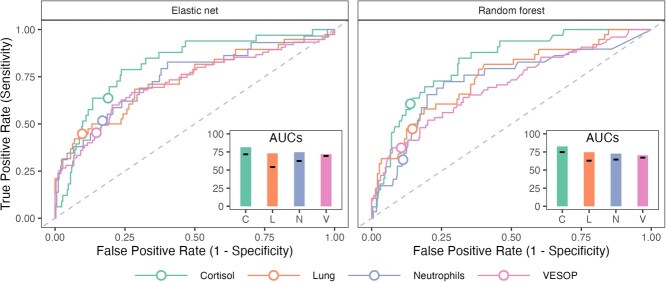
ROC curves for a subset of trait score models, illustrating the trade-off between sensitivity and specificity across thresholds used to convert predicted probabilities into binary classifications. All results show test set (out-of-sample) performance using models with optimized hyperparameters. Open circles denote sensitivity and specificity at the threshold that optimized F1 scores (harmonic mean between precision and recall) in the validation data. The dashed line $y=x$ indicates the performance of a random classifier. Inset plots display the area under the ROC curve (AUC); horizontal black markers show the AUCs of corresponding unoptimized models for comparison.

ROC curves and associated AUC values reflected these patterns (Fig. 7). AUCs for optimized elastic net models ranged from 72 (VESOP) to 85 (glucose), and 70 (VESOP) to 83 (cortisol) for random forest equivalents, reflecting fair to good predictive ability. Performance was slightly better for elastic net than random forest models (elastic net mean AUC = 78.2; random forest mean AUC = 75.0) and improved with hyperparameter optimization (elastic net $\varDelta$AUC = 13.4; random forest $\varDelta$AUC = 10.3).

## Discussion

DNAm patterns have by now been shown to be strongly predictive of chronological age across many wildlife species, and associations between DNAm-derived age acceleration and health outcomes, although still relatively new, are being increasingly reported (e.g. cardiovascular disease, (Levine *et al.,* 2018)). These findings strongly echo historical developments in human epigenetic research, which is more established and better resourced. The human epigenetic literature is an important resource for any discussion of the statistical considerations around wildlife epigenetic studies. Many of the methodological issues arising when studying humans also affect the analysis of wildlife data, while new methods in the human literature may be applicable or extendable to wildlife populations. Theoretically, there is no reason why methods developed for humans cannot be applied to wildlife. The essential input data—DNAm, age and health biomarkers—are the same and can, at least in principle, be sampled for many wildlife populations. However, there are substantial practical differences between human and wildlife studies, in terms of the volume and types of data that can be collected and the outcomes that are deemed useful.

### Differences between human and wildlife epigenetic studies

#### Data differences

The main difference between human and wildlife epigenetic data is that human health research benefits from the availability of large databases (e.g. data used in (Levine *et al.,* 2018)), whereas wildlife data generally come from separate studies from independent research groups where chronological age is often unknown. The available population is typically smaller, finding animals is labour- and cost-intensive, sampling may be opportunistic and subject to bias (Newediuk *et al.,* 2025), and collection of the samples involves invasive techniques (particularly for blood samples but also for skin). Wildlife sample sizes have typically been much smaller than those used to study human populations. While sample sizes are certain to increase as the cost and accessibility of DNAm processing become more favourable, the difficulty and cost of sample collection limits what is achievable, so that wildlife sample sizes will likely always be substantially smaller than those for humans.

There are methodological consequences to having relatively small sample sizes in wildlife epigenetic studies. A small sample size (i) limits the power to detect changes in methylation patterns and associations between methylation and external outcomes, e.g. epigenetic age and health outcomes: for example, with a sample size of 200 animals, a correlation must exceed 0.18 to be significant at $P<0.01$; with 10 000 samples a correlation of only 0.03 is needed; (ii) constrains methodological development, to some extent: for example, there have been recent attempts to construct epigenetic clocks using data-intensive deep learning approaches in human studies (Prosz *et al.,* 2024) and (iii) delays the curation of standard benchmarking datasets that exist for humans, e.g. reference catalogues for cell-type deconvolution, that require large sample size and diversity as well as rich phenotypic and clinical metadata.

Furthermore, there are many wildlife species but only one extant human species. Careful pooling of information across species, where appropriate, may counter some sample size issues. Current evidence suggests that predictions of chronological age within an animal population can be improved by including data from related species (Zoller *et al.,* 2025). However, the degree of improvement likely depends on many factors, including the size of the sample of interest, the size of the sample of related species, the tissue type, and the relatedness of the species. More multi-species applications will shed light on the potential benefits and risks of cross-species pooling.

Uncertainty in the chronological age of sampled individuals is an obvious problem for training epigenetic clocks, since age is the response variable. (Zoller *et al.,* 2025) state that “the importance of using known age animals… is paramount for the development of accurate epigenetic clock,” but the evidence for this is based on a comparison of two test populations that differ on other criteria (human care vs. wild) and is therefore not definitive. Our own results showed that accounting for uncertainty through the use of sample weights had no material effect on clock accuracy or associations with health. Epigenetic clocks are robust to a small amount of uncertainty in chronological ages, but uncertain ages become an increasing problem for training epigenetic clocks as the magnitude of the uncertainty increases (Mayne *et al.,* 2023; Newediuk *et al.,* 2025), as sample size drops, and if estimation errors are systematic rather than random. In these cases, the development of new methods that properly account for uncertainty in age estimates is an achievable target for future research.

#### Differences in research objectives

Perhaps the most important differences in objectives between wildlife and human epigenetic studies is that accurately predicting wildlife chronological age is more likely to be a goal in itself. Obtaining estimates of human chronological ages from DNAm data is commonly an intermediate step, because age is nearly always known.

In contrast, wildlife age is both commonly unknown and a key demographic input to models of population dynamics and many other ecological and biological processes. Human studies tend to focus on mid-to-late life effects on health and survival, and rely on assumptions of linear or log-linear methylation dynamics that are poor approximations of the rapid, non-linear changes that occur during early development (Vershinina *et al.,* 2021). In wildlife studies the interest is broader, across the whole lifespan (which varies between species), often with an interest in early life effects and effects on fecundity. Recently developed, second and third generation human clocks continue to focus on predicting adult mortality risk, typically excluding younger individuals (whose mortality is linked to developmental or acute causes rather than cumulative age-related decline), suggesting that wildlife-oriented approaches may need to take a different path, incorporating non-linear developmental trajectories to meet their objectives. Trade-offs between the accuracy of age predictions and the strength of associations between AARs and health outcomes are also likely to have important consequences for study design (see Section 5.2.3).

The primary attraction of wildlife epigenetic studies, especially those using remote skin biopsies, such as our case study, is that they can potentially provide indicators of certain health outcomes and a summary measure of animal health with a minimally invasive sampling protocol, while biopsies of tissue other than skin typically require animal capture and possibly sedation. This raises the possibility of monitoring the health status of wildlife populations, either over the long-term with repeated, e.g. annual, surveys or in response to exposure to a stressor or adverse event, e.g. an oil spill (Barratclough *et al.,* 2024). However, in most cases, the strength of associations between epigenetic outcomes, like age acceleration, and health outcomes, are at best moderate, limiting the power to detect changes or effects. This is also true for studies of human populations, but in those cases access to large sample sizes and diagnostic tools is a potential salve. Statistical power considerations will be important for any epigenetic study attempting to track animal health over time, as will careful definition and selection of any “healthy” or control groups used as a baseline for comparison, as is the case in human studies. For example, animals under human care are more likely to have longitudinal health records providing greater insight into the definition of case versus control.

#### Differences in methods

Given a particular methylation array, wildlife epigenetic studies produce the same quantitative data (i.e. a beta and $P$-value for each CpG site) as human studies and can, in principle, be analysed with the same methods. However, development of analytical approaches has exclusively been in the human domain. As a consequence, quantitative methodological developments in wildlife studies lag those in human studies. Epigenetic studies of wild animal populations have, to date, exclusively used FGECs to demonstrate that accurate prediction of chronological age is possible for many species (Lu *et al.,* 2023), and that age acceleration residuals are correlated with health outcomes (Barratclough *et al.,* 2024). However, some recent evidence suggests that FGECs might not always be suitable, e.g. for RRBS data as illustrated by (Simpson *et al.,* 2023). Similar models that directly predict traits (i.e. health outcomes) from DNAm inputs have also been applied (see Section 3.4.4), although these are much less common than in the human literature. As far as we are aware, no second-generation or other epigenetic clock models have been demonstrated for wildlife studies, likely because biomarker information is less commonly available. Methods like PhenoAge, GrimAge or ProbAge are all in principle directly applicable to wild animal studies, but empirical research is needed to establish their usefulness. We did not attempt the development of a PhenoAge-like clock for bottlenose dolphins due to a weak relationship between chronological age and mortality that was documented in the survival model in (Schwacke *et al.,* 2024), but the required biomarker information was available. Our application of ProbAge returned age accelerations that were more strongly associated with health outcomes than other epigenetic clocks. This mechanistic approach decouples the rate of change in methylation state (aging effects), from a systematic shift in global methylation (effects on the entire methylome due to stressor exposure or disease) (Dabrowski *et al.,* 2024) making it a better formulation for isolating biologically meaningful signals in health. Given that this approach takes the same input data as any first-generation clocks, retrospective analyses of other animal populations may be useful, particularly where external health outcomes are available.

### Statistical issues

#### Decoupling age and health prediction

Epigenetic clocks are interpreted as both (chronological) “age estimators,” and indicators of “innate biological processes” (Horvath and Raj, 2018) reflected in epigenetic age, but empirical studies have shown a trade-off between the ability to predict chronological age from DNAm data on one hand, and the ability to predict biological health from AARs on the other (Zhang *et al.,* 2019). In other words, clocks that predict age more accurately are worse at explaining health outcomes. The most plausible explanation for this (see supplementary material Fig. S1) is that improvements in chronological age accuracy arise when models have sufficient data or flexibility to include CpG sites that are not strongly correlated with aging, but are indicators of health conditions or behaviours that cause systematic changes in methylation patterns (Dabrowski *et al.,* 2024). Including these CpG sites allows clocks to compensate for (or “add back”) those differences between epigenetic and chronological age caused by the respective conditions and behaviours (Fig. S1). If these conditions and behaviours are included in the systematic component of the clock, they cannot then appear in the residual component that is used to assess health associations.

Our evidence was mixed but generally supported this trade-off. Models trained on control populations predicted chronological age less accurately overall than models trained on both control and exposed populations (Fig. 2d), but had AARs that were more strongly associated with health (Fig. 4). The lower overall accuracy of models trained on control populations occurs because they do worse at predicting the ages of animals in the exposed populations (Fig. 3). Intuitively, if one trains a clock on control animals, exposed animals’ ages will be poorly predicted, but residuals will be indicative of health. Conversely, training on both control and exposed animals means better predictions of exposed animals’ ages but less informative (about health) residuals. Log-linear transformations of age also improved age predictions (Fig. 3d) and reduced the number of statistically significant health associations (Fig. 5), but results differed from clock to clock. This may be because trade-offs only become clearer when prediction errors are very small, and ours are not. On balance, the current evidence points to a fundamental limit to what can be achieved with FGECs that try to predict both age and health, and suggests that these tasks should be decoupled. This also would seem to answer the question of “why epigenetic acceleration tracks with certain age-related disorders and not others” (Simpson and Chandra, 2021)—this is precisely what one would expect if some disorders affect methylation in non-age related CpGs and others not.

Decoupling age and health prediction seems especially important for wildlife studies, where age prediction is likely to be a direct goal. For example, one might use a FGEC for predicting age, a mechanistic model like ProbAge (Dabrowski *et al.,* 2024) for age-related health, and a trait score model for predicting specific phenotypes. FGECs already provide accurate age predictions for many species, and this accuracy will likely improve as more data and more flexible methods become accessible. However, caution needs to be used to avoid the trade-off described above. In our case study, the age acceleration metric produced by ProbAge was at least as effective as AARs from FGECs in terms of tracking animal health. Although more work is needed, this more mechanistic approach to age acceleration seems promising.

SGECs in theory avoid the trade-offs the FGECs face, and so can potentially be used to predict both age and health. However, they require the specification of biomarkers. For wildlife studies, this potentially adds substantial effort to data collection, and if biomarkers are only measurable in blood samples then this negates a key advantage of skin-based epigenetic studies—less invasive sampling that is both time- and cost-effective. Criteria for the selection of biomarkers have not been established, and will be somewhat dependent on the external health outcome of interest and varying pathophysiologies involved. None of this precludes the possibility of SGECs being applicable in wildlife studies, but there are methodological issues that would need attention, the answers to which may be species-specific. Any attempt to apply one of the SGEC methodologies to wildlife will likely have limited success if there is a weak relationship between age and mortality, because this leads to a wide range of ages that return similar mortality risks, and large uncertainty on any phenotypic age estimates. This requirement for a strong relationship between age and mortality is worth bearing in mind as this is not something that is generally known or acknowledged in human applications.

#### Which residuals? Choosing a baseline for health assessments

Age acceleration residuals have been widely used to investigate relationships between epigenetic age and health. There are different ways to define and use residuals, and these carry implications for downstream analyses. Residuals are nearly always defined either as differences between epigenetic and observed age (difference-based), or as the residuals from a model predicting epigenetic age from observed age (model-based). The rationale for using model-based residuals is to account for systematic relationships between chronological and epigenetic age, for example the underestimation of age for older individuals (Barratclough *et al.,* 2024). Note, however, that these relationships are based on the test set (i.e., the model is fit to the test data) and may or may not be present in training data (see supplementary material S1.5). Both difference-based and model-based approaches provide an absolute measure of age acceleration that assumes that the impact of a given residual (e.g., two years) is independent of the age of the individual. This may be justified when studying a cohort of similar ages, but studies comparing different life stages should consider whether a relative definition of age acceleration may be more appropriate (Hannum *et al.,* 2013), see also Supplementary Material S2 of (Dabrowski *et al.,* 2024)).

Difference-based and model-based AARs return the same rank ordering of age acceleration for all individuals of a certain age (i.e. conditional on chronological age), but rankings of age acceleration of individuals of different chronological ages may differ between approaches, as can the magnitude and sign of AARs. This is because there is a fixed frame of reference for difference-based AARs, observed age, whereas there is a different frame of reference for each modelling approach used to generate model-based AARs as it will have a different fitted line. Associations with health outcomes, whether measured by correlations or standardized regression coefficients, will therefore also differ. On average, one would expect model-based residuals to have lower variance than difference-based residuals and therefore to exhibit stronger associations with health outcomes, although this will depend on the dataset. We found stronger correlations with model-based residuals in 70% of tests across clocks, modelling conditions and health outcomes.

In general, it will be useful to consider what an AAR of zero means for any particular analysis. A natural interpretation is of an average healthy individual, but this leaves unspecified the baseline that “average” is compared to, and different residual approaches fill in this blank in different and sometimes obscure ways. First, consider the case where an existing clock is being applied to a new dataset. For difference-based residuals, an AAR of zero for an individual of age $a$ means that individual has the same conditional expected epigenetic age as individuals of age $a$ in whatever data was used to train the clock. This conditional mean will be informed by the chronological ages of all individuals in the training dataset, but a reasonable interpretation of an AAR of zero is “on average healthy relative to individuals of the same age in the training dataset.” This makes clear the dependence on the training sample, which for human studies is typically large and diverse, but may be much less so for wildlife studies. Model-based residuals on the other hand make the expected residual within the new sample zero, regardless of the health of the new sample and independent of the training data. An AAR of zero means “on average healthy relative to individuals of the same age in the new dataset.”

Now consider the case where AARs are being calculated for the same population used to construct the clock. Difference-based AARs retain their interpretation: an AAR of zero indicates an individual that is “on average healthy relative to individuals of the same age in the training dataset.” For model-based AARs, the previous interpretation—“on average healthy relative to individuals of the same age in the new dataset”—no longer makes sense because there is only one set of individuals. This confusion arises because model-based AARs use an individual’s epigenetic age, when they are part of the test set, to estimate the relationship between chronological and epigenetic age and therefore to adjust for any systematic biases. This means that each individual appears twice: once as training data and once as test data. But any true systematic biases should have been detected during the process of clock construction, and fitting an additional model to test predictions runs the risk of overfitting—this would certainly be true of predictions obtained by back-transforming model-based AARs. This will be especially important if AARs were used to predict health outcomes. Cross-fitting or double machine learning provides a potential solution to this problem (Kennedy, 2024).

#### Prediction or explanation?

Prediction and explanation are two distinct objectives with consequences for model choice, validation and interpretation (Breiman, 2001). Prediction aims to optimize performance on unseen data—accurately forecasting outcomes based on input features—whereas explanation seeks to uncover the underlying mechanisms or relationships among variables, often with an emphasis on statistical association or causality.

From a modelling perspective, predictive tasks are generally focused on empirical performance and agnostic about the underlying data-generating process. Highly flexible models are often preferred for their ability to capture complex, non-linear patterns in data, even when the reasons underlying their predictions are unclear. To date, use of elastic net regression to predict age from methylation data has been ubiquitous, with some arguing that “alternative statistical methods are not likely to lead to substantial improvements” (Horvath and Raj, 2018). However, evidence from many fields, including “small $n$, large $p$” settings, suggests that as sample sizes become bigger it will be hard to compete with deep neural networks on predictive accuracy. Where prediction is the aim, best practices in machine learning should be followed—these are summarized for epigenetic applications in Box 6 of (Yousefi *et al.,* 2022). They include choosing a training sample representative of, and therefore expected to generalise to, the broader population to which the model will be applied; splitting data into training, test and potentially validation samples and maintaining these splits throughout the model building process to avoid data leakage (possibly by resampling where sample sizes are too small for independent sets); and assessing out-of-sample performance using established metrics (e.g. accuracy, precision-recall curves, AUC) in a clearly demarcated test set. Examining performance within subcategories e.g. age classes, may provide insights into clock performance that are otherwise missed by aggregated assessments. While it is common practice to leave model hyperparameters such as the elastic net mixing parameter $\alpha$ at default values when constructing FGECs, best practice is to select hyperparameter values on the basis of performance on a validation set. Our results showed little difference between optimized and unoptimized clocks, and were also ambiguous about the value of pre-filtering CpG sites.

In contrast, explanatory tasks focus on understanding the data-generating process with models that yield identifiable, interpretable parameters that correspond to clearly defined conditional relationships, such as marginal or partial effects, e.g. generalized linear models, or hierarchical models. Under model-specific assumptions, e.g. linearity, independence and homoscedasticity, estimates of model parameters and their associated uncertainty allow researchers to draw inferences about the direction, strength and significance of relationships, and under additional assumptions these coefficients may be interpreted causally. Problems related to assumption violations, such as multi-collinearity, confounding and model misspecification, negatively impact explanatory models by causing biased parameter estimates or inflated standard errors that undermine the validity of conclusions drawn about underlying mechanisms, but are only of concern for prediction tasks to the extent that they reduce out-of-sample accuracy.

Models explaining epigenetic mechanisms with methylation as a (possibly multivariate) response variable need to choose an appropriate distributional form for the response (see Section 3.2). Methylation data are a ratio of counts forming a proportion, and can be modelled using beta or gamma-gamma distributions. Selection of distributional form is currently a pragmatic decision relating to available software for model building, and model diagnostics for this choice remain underdeveloped. Modelling methylation profiles as a multivariate response, although technically challenging because of the high dimensionality of the response, provides an appealing way of accounting for correlations between different CpG sites, potentially circumventing problems of multicollinearity that plague inference about influential CpG sites when methylation is a predictor.

Confounding is an ever-present problem for models seeking to describe the effect of epigenetic differences on outcomes of interest, for example health or age. A confounding variable is one that influences both predictor and response variables, and creates biased estimates of the effect of the predictor—a phenomenon known as omitted variables bias (Wilms *et al.,* 2021)—and spurious associations between predictor and response if not properly accounted for. For models predicting age from methylation data, many diseases are associated with both methylation and age and thus are potential confounders. For models investigating relationships between methylation and health, many diseases potentially associated with changes in methylation are also associated with other variables that themselves affect methylation, e.g. cell-type composition.

To our knowledge, no epigenetic study of wild animal populations has adjusted for confounders like cell-type composition, and a lack of reference catalogues means that there are limited means for doing so. Testing reference-free approaches, and developing reference catalogues within and across species, are areas needing further research. Epigenetic studies of wild animal populations, which to date have largely not considered explanatory mechanisms and have therefore avoided the problem of confounding present in the human setting, face perhaps a bigger challenge because potential confounders are both less likely to be known and less likely to be measurable during sampling. Accounting for missing confounding variables is likely to be an important topic for studies trying to understand epigenetic associations in both humans and animals. Causal inference (Ding and Li, 2018, e.g.) provides various tools for doing so, and some of these, e.g. latent variable models, have been used in the epigenetic literature to adjust for unobserved cell-type proportions (Houseman *et al.,* 2014). They may also be applicable or adaptable for more general inference on epigenetic associations (Liu *et al.,* 2022).

## Conclusions

In this review, we present the research objectives of wildlife epigenetic studies, review current and potential analytical approaches, and discuss their implications for making inferences from wildlife DNAm data. Epigenetic studies of wildlife populations lag behind human studies in terms of application and methods development. There are fundamental differences between human and wildlife applications of epigenetic data analyses in terms of data volume, the variables that can be measured, research objectives and analytical approaches: wildlife datasets are tiny in comparison, age and clinical information are commonly unknown, predicting age and health status are a central goal, and longitudinal monitoring of individuals is rare. There is a trade-off between predicting age and health, which means that a single model cannot do both accurately: residuals from models constructed to predict age well should not be interpreted as health indicators. Furthermore, the interpretation of residuals is different depending on the way they are generated and needs to be carefully considered if residuals are used in downstream analyses. Acknowledging that the analysis of age and health needs to be decoupled is particularly important in the wildlife setting due to frequently unknown age and varying lifespans, and the importance of age in broader ecological and demographic analyses. Lastly, confounding between age and health makes this decoupling hard and is a topic for future methodological development.

## Supplementary Material

Web_Material_coaf091

## Data Availability

The data used for illustration in this paper come from multiple primary sources; to make it possible for readers to run the analyses presented here we provide all code and code to generate a synthetic version of data in supporting files. As such, the data provided do not reproduce the results in this paper but can be used to run the code, found here https://doi.org/10.5281/zenodo.17886951, and apply it to their own datasets.
