# Supplementary material for: Methods for analysing wildlife DNA methylation data
Source: Conserv Physiol. 2026 Feb 25;14(1):coaf091. doi: 10.1093/conphys/coaf091 (PMC12935468; doi:10.1093/conphys/coaf091)
Supplement: Web_Material_coaf091 [file web_material_coaf091.zip › Photopoulou et al_SuppMat.pdf]

Supplementary Material

The material presented here is a supplement to "Methods for analysing wildlife DNA methylation data" by Theoni Photopoulou, Ian Durbach, Enrico Pirotta, Ashley Barratclough, Lori H. Schwacke, Ryan Takeshita, Gina Himes Boor, Catriona M. Harris, Peter L. Tyack, and Len Thomas (2026). Code used to carry out this analysis can be found here `https://doi.org/10.5281/zenodo.17886951`.

S1.1 Cell-type deconvolution

The general deconvolution problem (i.e., determining which cell types make up a sample and in what proportion) can be stated as $\boldsymbol{M} = \boldsymbol{W}\boldsymbol{R}$ where $\boldsymbol{M}$ is the matrix of observed DNAm profiles as above, $\boldsymbol{W}$ is a $n \times c$ matrix of sample-specific cell-type proportions (n samples, c cell types), and $\boldsymbol{R}$ is a $c \times p$ matrix of reference DNAm profiles denoting mean DNAm values within each cell type. Methods assuming $\boldsymbol{R}$ is known (from some external data source) and estimating $\boldsymbol{W}$ are called *reference-based*; those simultaneously estimating both $\boldsymbol{R}$ and $\boldsymbol{W}$ are called *reference-free* methods. Benchmarking studies suggest reference-based approaches are more precise but may be more sensitive to unknown confounding effects than reference-free approaches (Teschendorff and Relton, 2018; De Ridder et al., 2024). The end goal is inclusion of $\boldsymbol{W}$ as a covariate in downstream analyses, controlling for heterogeneity in cell types.

Two major themes in cell deconvolution research are 1) developing appropriate statistical approaches for deconvolution, and 2) how to construct reference sets for reference-based approaches. Many methods for estimation of $\boldsymbol{W}$ and $\boldsymbol{R}$ exist, including multivariate regression, support vector regression, with or without principal component analysis, and constrained projection/quadratic programming. Methods are available that correct for erroneous methylation values using expectation maximisation (*EMeth* (Zhang et al., 2021)) or hidden Markov models (*PRISM* (Lee et al., 2019)). A main discriminating feature is whether $\boldsymbol{W}$ is constrained or not. If not, post-hoc fixes are usually applied to produce proportions.

Construction of reference sets is often constrained by practical considerations such as budget and sample size, but in general these sets should be representative of the samples in $\boldsymbol{M}$ in terms of e.g., age, sex, health, etc. Precision of estimators of $\boldsymbol{W}$ can also be improved by a construction of $\boldsymbol{R}$ that minimises collinearity (Newman et al., 2015; Teschendorff and Zheng, 2017).

S1.2 Methods for sample collection and processing

Methylation data were obtained from 476 skin samples collected from 429 unique bottlenose dolphins. Of these, 426 came from 389 free-ranging animals from eight management stocks (four of Tamanend's bottlenose dolphin (*Tursiops erebennus*), four of common bottlenose dolphin (*Tursiops truncatus*) sampled as part of catch-and-release health assessment studies or remote biopsy sampling), while 50 samples are from 50 individuals at the U.S. Navy Marine Mammal Program (Barratclough et al., 2024).

Samples from free-ranging animals were obtained between 1991 and 2022 and stored in a 20% dimethyl sulfoxide (DMSO) solution saturated with sodium chloride. DNA had been previously extracted from skin for some of the samples ($n = 299$) using a phenol-chloroform protocol as described in Rosel and Block (1996) or using a DNeasy Blood and Tissue Kit (Qiagen) following

manufacturer's protocols and was archived at the NOAA Southeast Fisheries Science Center, Marine Mammal Molecular Genomics Laboratory in Lafayette, Louisiana (Rosel and Block, 1996). DNA was extracted from skin for the remaining free-ranging samples ($n = 127$) using a DNeasy Blood and Tissue Kit (Qiagen, Hilden, Germany) as above. DNA quality was assessed via gel electrophoresis and DNA concentration was determined using a Qubit4 fluorometer (Invitrogen) or a Hoefer DyNAQuant 200 fluorometer (GE Healthcare). For each sample, approximately 300 ng of DNA was loaded into a 96-well plate and dried down using a SpeedVac (Savant); when dry, plates were sealed and submitted to The Clock Foundation (Torrance, CA) for DNA methylation analysis. DNA for Navy samples were extracted using the DNeasy Blood and Qiagen Tissue Kit and plated at The Clock Foundation.

DNA methylation was measured using a custom mammalian methylation array (HorvathMammalMethylChip40) with 37,492 CpG sites (Arneson et al., 2022). A beta value was created for each CpG site by obtaining the ratio of the fluorescence intensity of a methylated probe for that specific site to the total overall probe intensity (Du, 2010). The SeSAMe pipeline was used to normalise the raw data and produce an estimate of the degree of methylation, for each CpG site for each individual (Zhou et al., 2018). A detection p-value was also produced from the pipeline to accompany each normalised beta value, which can be interpreted as the probability that the signal overlaps with the background distribution of fluorescence (Zhou et al., 2018). Beta values range from zero to one, with zero indicating no methylation at that CpG site (Zhou et al., 2018).

S1.3 Decoupling health and age predictions

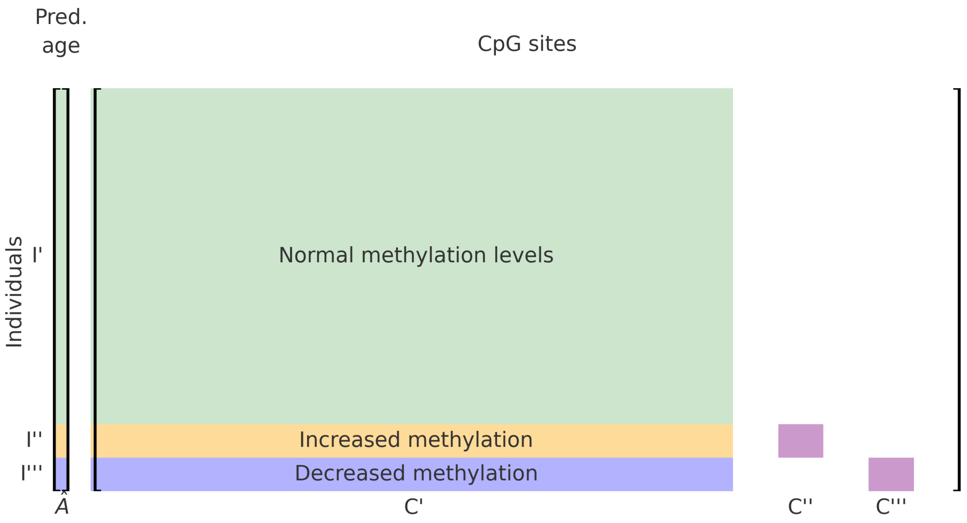

Figure S1. Why clocks that predict age more accurately are worse at explaining health outcomes. Methylation at age-related CpG sites (C') accurately predicts chronological age ($\hat{A}$) for the majority "healthy" population (I'), but is higher for those with an adverse health condition (I'') and lower for those with a positive condition (I'''). With small sample sizes and simple (e.g., linear) models, only associations between age and methylation at C' are detected and included in predictions. Age is over- and underestimated for those in I'' and I''' respectively, and residuals are diagnostic of the health conditions that caused these systematic errors. However if these conditions also affect methylation at CpG sites unrelated to aging (C'' and C'''), then larger sample sizes and more powerful predictive models can tease out these weaker associations between chronological age and methylation at C'' and C''', and use them to improve age predictions in I'' and I''' respectively.

S1.4 Supplementary Methods

S1.4.1 Data preprocessing (`00_data_prep.R`)

Cross-validation setup

We implemented 10-fold cross-validation with stratification by site. Individuals were classified as either from control or non-control sites. The cross-validation partitioning was conducted separately within the control and non-control groups, and individuals were then assigned cyclically such that each fold contained approximately equal proportions of control and non-control individuals. Individuals (rather than samples) were partitioned into $K = 10$ outer folds so that repeated biopsies never crossed folds. This ensured that observations from the same animal did not appear simultaneously in both training and testing sets. A fixed random seed was used to ensure full reproducibility.

For each fold i, all individuals within that fold were designated as the test set. The remaining individuals were then randomly divided into training (85%) and validation (15%) sets. This partitioning was performed separately for each fold, but again independently within control status groups to maintain the same proportional representation.

One of the aims of our paper is to assess the effect of fitting models to individuals from control sites only, rather than from both control and non-control sites. To ensure that differences are due to differences in sites, rather than differences in sample size, we constructed a second "reduced size" dataset containing the same number of individuals as in the full controls-only dataset, but drawn from both control and non-control dataset.

To assign folds to the reduced dataset, we randomly draw C/K individuals from each fold, where C is the number of control individuals in the full dataset and $K = 10$ is the number of folds. for the test set, ensuring equal size across folds. All individuals within reduced fold i were designated as the test set, with the remaining control individuals in other folds randomly divided into training (85%) and validation (15%) sets associated with fold i, and the same done for non-control individuals.

Age transformations

For each dolphin with chronological age A (years) we considered three transformations:

$$
\begin{aligned}
T_{\text{lin}}(A) &= A, \\
T_{\log}(A) &= \log\{\max(A, 0.01)\}, \\
T_{\text{LL}}(A \mid m) &= \begin{cases} \log\left(\dfrac{A+k}{m+k}\right), & A < m, \\ \dfrac{A-m}{m+k}, & A \geq m, \end{cases}
\end{aligned}
$$

with m = `loglin_sexm` = 15 yr is the age of sexual maturity and offset $k = 0.01$. The inverse of T_{LL} is

$$
T_{\text{LL}}^{-1}(z) = \begin{cases} (m+k)\exp(z) - k, & z < 0, \\ (m+k)z + m, & z \geq 0, \end{cases}
$$

Hyperparameters

All first-generation clock models were trained over all combinations of parameters shown in Table 1, except that when `controls_only=TRUE`, `reduce_size` was fixed to `FALSE`. Feature screening to the top n_{CpG} sites (ranked by absolute Pearson correlation with the transformed age) was conducted separately inside every training fold to avoid information leakage.

hyperparameter	Candidate values
α (elastic net mixing)	$\{0.01, 0.1, 0.2, \dots, 0.9, 0.99\}$
n_{CpG} (features retained)	$\{500, 1000, 2000, 31157\}$
`use_weights`	{`TRUE`, `FALSE`}
`controls_only`	{`TRUE`, `FALSE`}
`reduce_size`	{`TRUE`, `FALSE`}
`loglin_age`	{`"lin"`, `"log"`, `"loglin"`}
`rf_mtry` (random forest only)	$\{1/6, 1/3, 1/2\}$

Table 1: Grid searched by `01_run_all.R`. When `controls_only=TRUE`, `reduce_size` was fixed to `FALSE`.

S1.4.2 Model fitting (`01_run_all.R`)

The wrapper script `01_run_all.R` fits all clock (elastic net, random forest, ProbAge) and trait score (elastic net, random forest) models configurations for each hyperparameter combination, generates predictions from these models for the training, validation, and test subsets, and saves these for downstream processing.

Elastic net first-generation clocks (`fgecs.R`)

For each hyperparameter combination θ from Table 1 we trained an elastic net by minimizing

$$\min_{\beta_0,\boldsymbol{\beta}} \frac{1}{2n} \sum_{j\in\mathcal{T}} w_j \big[T(A_j) - \beta_0 - \mathbf{M}_j^\top \boldsymbol{\beta}\big]^2 + \lambda \left\{ (1-\alpha)\frac{\|\boldsymbol{\beta}\|_2^2}{2} + \alpha\|\boldsymbol{\beta}\|_1 \right\},$$

where $\mathcal{T}$ indexes the training rows for the fold, w_i equals the optional age weight, and $T(\cdot)$ is one of the transforms above. Predictors were optionally filtered to the $n_{\text{CpG}} \in \{500, 1000, 2000, 31157\}$ sites with largest absolute Pearson correlation with $T(A)$ inside each training set. Models were fit with `glmnet` using the fold-specific training/validation splits rather than `glmnet`'s internal cross-validation, and predictions were mapped back to chronological age through T^{-1}.

Random forest clocks (`fgecs.R`)

Random forests received the same training responses and screened predictor sets as for the elastic nets. Each forest used 1,000 trees, and the number of candidate CpGs at a split was $\max\{500, \lfloor r\,p \rfloor\}$ with $r \in \{1/6, 1/3, 1/2\}$ and p the count of retained CpGs. Predicted values were inverse-transformed via T^{-1}.

ProbAge site-level likelihood (`probage.R`)

Methylation at each CpG site i and individual j was assumed Beta-distributed with mean

$$\mu_i(t_j) = \eta_i + e^{-\omega_i t_j}\big(p_i - \eta_i\big),$$

and variance

$$\sigma_i^2(t_j) = \frac{\eta_i(1-\eta_i)}{N_i} + e^{-\omega_i t_j}\left[\frac{(1-p_i)\eta_i^2 + p_i(1-\eta_i)^2 - \eta_i(1-\eta_i)}{N_i}\right] + e^{-2\omega_i t_j}\left[\frac{c_i}{N_i^2} - \frac{(1-p_i)\eta_i^2 + p_i(1-\eta_i)^2}{N_i}\right].$$

Here, η_i denotes the asymptotic (steady-state) methylation proportion, p_i the initial methylation level, ω_i the site-specific transition rate, and N_i, c_i control dispersion.

Site parameters $\boldsymbol{\theta}_i = (\eta_i, \omega_i, p_i, N_i, c_i)$ were estimated by maximizing the weighted log-likelihood

$$\ell_i = \sum_{j\in\mathcal{T}} w_j \,\log B\big(m_{ij}(t_j);\ \mu_i(t_j),\ \sigma_i^2(t_j)\big),$$

using `optim`, after restricting to the n_{CpG} CpG sites with the strongest absolute correlation with age and excluding sites with $\omega_i > 1000$ or $\eta_i, p_i > 0.95$.

Conditional on the retained site parameters, individual-specific acceleration and bias parameters (α_j, β_j) were estimated by re-optimizing the likelihood under the adjusted parameters $\omega_i^{(\text{adj})} = \omega_i\, e^{\alpha_j}$, $\eta_i^{(\text{adj})} = \text{logit}^{-1}\big(\text{logit}(\eta_i) + \beta_j\big)$, and $p_i^{(\text{adj})} = \text{logit}^{-1}\big(\text{logit}(p_i) + \beta_j\big)$, keeping all other terms fixed. The parameters α_j and β_j were interpreted as individual-level acceleration and bias effects.

Health-trait prediction (healthmodels.R)

Health models mirrored the FGEC design but substituted binary outcomes, including the Veterinary Expert System for Outcome Prediction (VESOP) failure flag (< 0.925) and individual biomarker flags. Elastic nets minimised the binomial deviance

$$\min_{\beta_0,\boldsymbol{\beta}} -\frac{1}{n}\sum_{j\in\mathcal{T}} y_j(\beta_0 + \mathbf{M}_j^\top\boldsymbol{\beta}) - \log(1+e^{\beta_0+\mathbf{M}_j^\top\boldsymbol{\beta}}) + \lambda\left\{(1-\alpha)\frac{\|\boldsymbol{\beta}\|_2^2}{2} + \alpha\|\boldsymbol{\beta}\|_1\right\},$$

where $(\beta_0 + \mathbf{M}_j^\top\boldsymbol{\beta})$ and $y_i \in \{0,1\}$.

S1.4.3 Postprocessing

Script `02_postprocessing.R` computed validation-set median absolute error within each configuration of hyperparameters and selected the hyperparameters minimizing this error. These optimal settings were then evaluated on the held-out test folds to summarise out-of-sample performance (MAE, R^2, correlation) and to provide the prediction sets used in all figures and tables. Script `03_calc_predictions_and_aars.R` extracted fold-specific test predictions to produce one DNAm age per dolphin, merged those estimates with predictions from existing clocks, and constructed two simple ensembles (i) the unweighted mean across five models (optimised elastic net and random forest FGECs, and the three existing clocks) and (ii) a weighted mean of the same five models. Weights were calculated by fitting a linear regression model to the training-set predicted ages from all five clocks to estimate their optimal combination for predicting chronological age. These coefficients were then applied to the corresponding test-set predictions to yield an out-of-sample ensemble prediction.

Let $\widehat{A}_j$ denote the out-of-sample DNAm age prediction for individual j, and let A_j denote their chronological age. Age-acceleration residuals were computed as (i) $\mathrm{AAR}_j^{\mathrm{diff}}$: raw differences $\widehat{A}_j - A_j$, (ii) $\mathrm{AAR}_j^{\mathrm{lin}}$: residuals from $A_j = \gamma_0 + \gamma_1\widehat{A}_j + \varepsilon_j$, and (iii) $\mathrm{AAR}_j^{\mathrm{gam}}$: residuals from $A_j = f(\widehat{A}_j) + \varepsilon_j$ with f a thin-plate spline (`mgcv::gam`, $k = 4$). The first retains the raw prediction bias, the second enforces linear decorrelation from age, and the third allows feature-dependent nonlinear calibration. All three rely solely on out-of-sample $\widehat{A}_i$. Downstream plotting scripts (`04_plots_tables.R`, `05_plots_health.R`) therefore display only test-set metrics.

S1.5 Calculation of Age Acceleration Residuals (AAR)

The motivation for the use of the adjusted $\mathrm{AAR}_j^{\mathrm{lin}}$ or $\mathrm{AAR}_j^{\mathrm{gam}}$ is that out-of-sample epigenetic age estimates often exhibit systematic deviation from the identity line (e.g. underestimation of age at older ages). For age acceleration defined as $\mathrm{AAR}_j^{\mathrm{diff}} = \widehat{A}_j - A_j$ these biases induce correlation between AARs and age and potentially inflate associations between AARs and health outcomes. Post-processing the out-of-sample predictions by using a calibration (linear) regression to remove this confounding has become standard practice (e.g. Horvath (2013); Lu et al. (2023)). This calibration removes linear bias and ensures $\mathrm{AAR}^{\mathrm{lin}}$ and A are uncorrelated.

To clarify the implementation of calibrating regressions, we briefly describe three scenarios. Let $\boldsymbol{M} \in \mathbb{R}^{n\times p}$ denote DNA methylation measurements for n individuals across p CpG sites. An epigenetic clock is learned via regularised regression (e.g. elastic net), yielding coefficients $\hat{\beta}_0, \hat{\boldsymbol{\beta}} \in \mathbb{R}^p$. For an individual j, the predicted epigenetic age is $\hat{A}_j = \hat{\beta}_0 + \boldsymbol{M}_j^\top\hat{\boldsymbol{\beta}}$. The three scenarios we consider are

1. The same samples are used to train the clock (estimate $\boldsymbol{\beta}$) and perform the calibration (estimate $\boldsymbol{\gamma}$), and there is no independent holdout set (as in our case study);

2. The same samples used to train the clock and perform the calibration, and there is an independent test set;

3. Epigenetic age estimates are obtained using an existing clock.

When the same dataset is used for both training and AAR calculation and no independent holdout set is available, we obtain out-of-sample predictions using K-fold cross-validation. Let $\mathcal{F}_k$ be the kth fold and $\mathcal{T}_k = \{1, \ldots, n\} \setminus \mathcal{F}_k$ be the training set associated with fold k. For $j \in \mathcal{F}_k$, predicted age is computed using the model trained on $\mathcal{T}_k$:

$$\hat{A}_j^{(\mathrm{CV})} = \hat{\beta}_0^{(k)} + \boldsymbol{M}_j^\top \hat{\boldsymbol{\beta}}^{(k)}, \quad j \in \mathcal{F}_k.$$

These cross-validated predictions are then adjusted via regression,

$$\hat{A}_j^{(\mathrm{CV})} = \gamma_0 + \gamma_1 A_j + \varepsilon_j,$$

and

$$\mathrm{AAR}_j^{\mathrm{lin}} = \hat{\varepsilon}_j = \hat{A}_j^{(\mathrm{CV})} - (\hat{\gamma}_0 + \hat{\gamma}_1 A_j)$$

Using out-of-fold predictions prevents overfitting from influencing the AAR values. In contrast, if in-sample age estimates were regressed on true age, one would typically observe little to no adjustment (especially in cases of overfitting) and therefore persistent correlation between $\mathrm{AAR}^{\mathrm{lin}}$ and A (Figure S2).

If the clock is trained using a subset $\mathcal{T} \subset \{1, \ldots, n\}$, and AARs are evaluated in an independent holdout set $\mathcal{H}$ (with $\mathcal{T} \cap \mathcal{H} = \emptyset$), model coefficients $\hat{\beta}_0$ and $\hat{\boldsymbol{\beta}}$ are estimated using only $\mathcal{T}$. Predicted ages for individuals in the holdout set are then computed as

$$\hat{A}_i^{(\mathrm{holdout})} = \hat{\beta}_0 + \boldsymbol{M}_j^\top \hat{\boldsymbol{\beta}}, \quad j \in \mathcal{H}.$$

Bias adjustment is then performed as before.

$$\hat{A}_j^{(\mathrm{holdout})} = \gamma_0 + \gamma_1 A_j + \varepsilon_j,$$

and

$$\mathrm{AAR}_j^{\mathrm{holdout}} = \hat{\varepsilon}_j = \hat{A}_j^{(\mathrm{holdout})} - (\hat{\gamma}_0 + \hat{\gamma}_1 A_j)$$

This approach ensures that both age prediction and AAR estimation are entirely out-of-sample.

When using an published epigenetic clock, the model coefficients have already been estimated using external data. Denote these parameters by $\hat{\beta}_0$ and $\hat{\boldsymbol{\beta}}$. For individual j, the predicted epigenetic age is

$$\hat{A}_j^{(\mathrm{pub})} = \hat{\beta}_0 + \boldsymbol{M}_j^\top \hat{\boldsymbol{\beta}},$$

which is inherently out-of-sample with respect to the current cohort. To correct systematic bias one regresses predicted epigenetic age on chronological age as before,

$$\hat{A}_j^{(\mathrm{pub})} = \gamma_0 + \gamma_1 A_j + \varepsilon_j,$$

and

$$\mathrm{AAR}_j^{\mathrm{pub}} = \hat{\varepsilon}_j = \hat{A}_j^{(\mathrm{pub})} - (\hat{\gamma}_0 + \hat{\gamma}_1 A_j)$$

In all three cases, AARs are calculated using only out-of-sample $\widehat{A}_j$ to ensure that age acceleration is uncorrelated with age and so to remove the effect of age on associations between age association and health. AARs calculated in this way are appropriate for downstream association analyses but should not be used to assess predictive performance of clocks, which should be evaluated on $\widehat{A}_j$ directly.

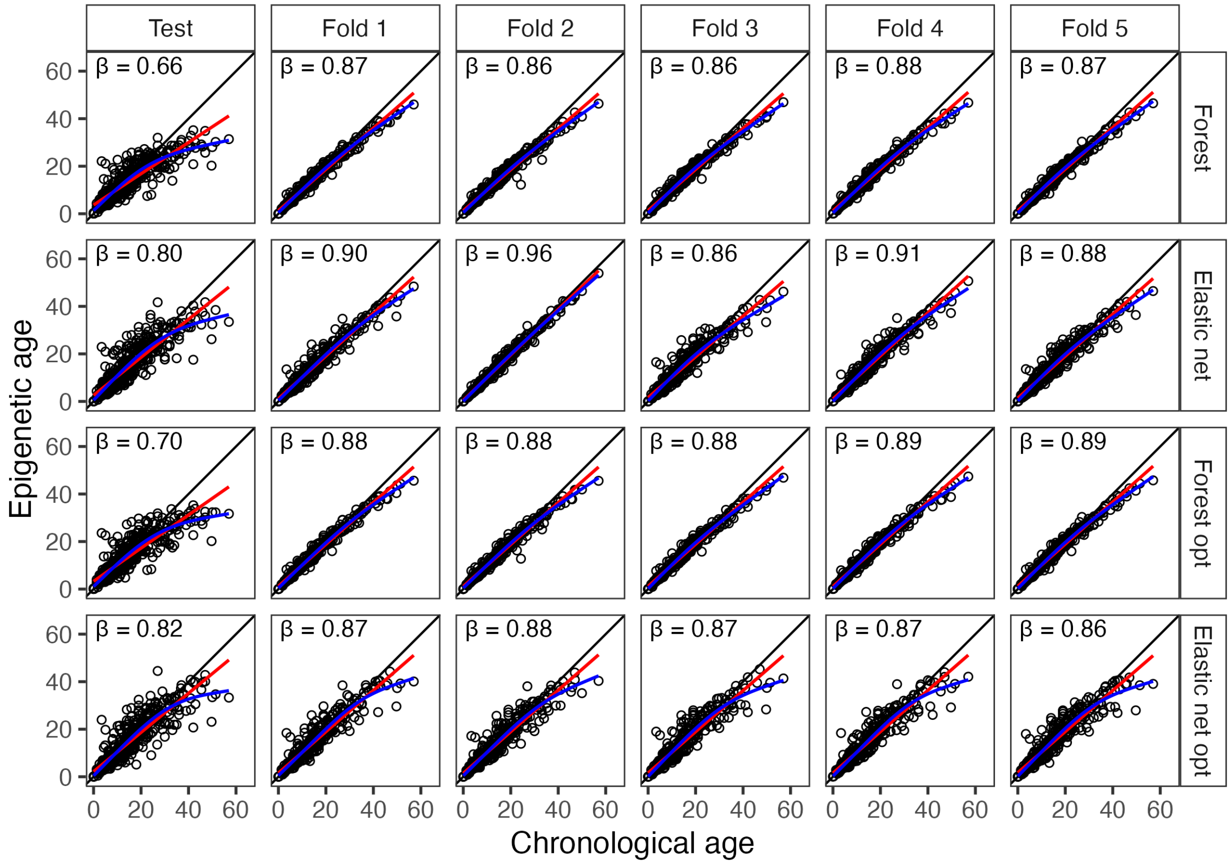

Figure S2. Out-of-sample estimates of chronological age (first column, "Test") show consistently greater systematic bias (underestimating chronological age for older individuals) than in-sample estimates (columns 2-6). Within-fold in-sample estimates are shown for five of the ten training folds used. Points show chronological and epigenetic age for selected clocks, with log-linearly transformed age, models trained on all individuals, sample weights not used. Solid black, red, and blue lines denote the line $y = x$ and curves from fitted linear and generalised additive models respectively. The slope coefficient for the fitted linear model is shown in the top left of each panel, and is consistently closer to one when in-sample estimates are used.

References

Arneson, A., Haghani, A., Thompson, M. J., Pellegrini, M., Kwon, S. B., Vu, H., Maciejewski, E., Yao, M., Li, C. Z., Lu, A. T., Morselli, M., Rubbi, L., Barnes, B., Hansen, K. D., Zhou, W., Breeze, C. E., Ernst, J., and Horvath, S. (2022). A mammalian methylation array for profiling methylation levels at conserved sequences. *Nature Communications*, 13(1):783.

Barratclough, A., Takeshita, R., Thomas, L., Photopoulou, T., Pirotta, E., Rosel, P. E., Wilcox Talbot, L. A., Vollmer, N. L., Wells, R., Smith, C. R., Rowles, T. K., Horvath, S., and Schwacke, L. (2024). Estimating age and investigating epigenetic changes related to health across multiple bottlenose dolphin populations. *Biological Conservation*, 293:110570.

De Ridder, K., Che, H., Leroy, K., and Thienpont, B. (2024). Benchmarking of methods for DNA methylome deconvolution. *Nature Communications*, 15(1):4134.

Du, P. e. a. (2010). Comparison of beta-value and m-value methods for quantifying methylation levels by microarray analysis. *BMC Bioinformatics*, 11:587.

Horvath, S. (2013). DNA methylation age of human tissues and cell types. *Genome Biology*, 14(10):3156.

Lee, D., Lee, S., and Kim, S. (2019). Prism: methylation pattern-based, reference-free inference of subclonal makeup. *Bioinformatics*, 35(14):i520–i529.

Lu, A. T., Fei, Z., Haghani, A., Robeck, T. R., Zoller, J. A., Li, C. Z., Lowe, R., Yan, Q., Zhang, J., Vu, H., Ablaeva, J., Acosta-Rodriguez, V. A., Adams, D. M., Almunia, J., Aloysius, A., Ardehali, R., Arneson, A., Baker, C. S., Banks, G., Belov, K., Bennett, N. C., Black, P., Blumstein, D. T., Bors, E. K., Breeze, C. E., Brooke, R. T., Brown, J. L., Carter, G. G., Caulton, A., Cavin, J. M., Chakrabarti, L., Chatzistamou, I., Chen, H., Cheng, K., Chiavellini, P., Choi, O. W., Clarke, S. M., Cooper, L. N., Cossette, M. L., Day, J., DeYoung, J., DiRocco, S., Dold, C., Ehmke, E. E., Emmons, C. K., Emmrich, S., Erbay, E., Erlacher-Reid, C., Faulkes, C. G., Ferguson, S. H., Finno, C. J., Flower, J. E., Gaillard, J. M., Garde, E., Gerber, L., Gladyshev, V. N., Gorbunova, V., Goya, R. G., Grant, M. J., Green, C. B., Hales, E. N., Hanson, M. B., Hart, D. W., Haulena, M., Herrick, K., Hogan, A. N., Hogg, C. J., Hore, T. A., Huang, T., Izpisua Belmonte, J. C., Jasinska, A. J., Jones, G., Jourdain, E., Kashpur, O., Katcher, H., Katsumata, E., Kaza, V., Kiaris, H., Kobor, M. S., Kordowitzki, P., Koski, W. R., Krützen, M., Kwon, S. B., Larison, B., Lee, S. G., Lehmann, M., Lemaitre, J. F., Levine, A. J., Li, C., Li, X., Lim, A. R., Lin, D. T. S., Lindemann, D. M., Little, T. J., Macoretta, N., Maddox, D., Matkin, C. O., Mattison, J. A., McClure, M., Mergl, J., Meudt, J. J., Montano, G. A., Mozhui, K., Munshi-South, J., Naderi, A., Nagy, M., Narayan, P., Nathanielsz, P. W., Nguyen, N. B., Niehrs, C., O'Brien, J. K., O'Tierney Ginn, P., Odom, D. T., Ophir, A. G., Osborn, S., Ostrander, E. A., Parsons, K. M., Paul, K. C., Pellegrini, M., Peters, K. J., Pedersen, A. B., Petersen, J. L., Pietersen, D. W., Pinho, G. M., Plassais, J., Poganik, J. R., Prado, N. A., Reddy, P., Rey, B., Ritz, B. R., Robbins, J., Rodriguez, M., Russell, J., Rydkina, E., Sailer, L. L., Salmon, A. B., Sanghavi, A., Schachtschneider, K. M., Schmitt, D., Schmitt, T., Schomacher, L., Schook, L. B., Sears, K. E., Seifert, A. W., Seluanov, A., Shafer, A. B. A., Shanmuganayagam, D., Shindyapina, A. V., Simmons, M., Singh, K., Sinha, I., Slone, J., Snell, R. G., Soltanmaohammadi, E., Spangler, M. L., Spriggs, M. C., Staggs, L., Stedman, N., Steinman, K. J., Stewart, D. T., Sugrue, V. J., Szladovits, B., Takahashi, J. S., Takasugi, M., Teeling, E. C., Thompson, M. J., Van Bonn, B., Vernes, S. C., Villar, D., Vinters, H. V., Wallingford, M. C., Wang, N., Wayne, R. K., Wilkinson,

G. S., Williams, C. K., Williams, R. W., Yang, X. W., Yao, M., Young, B. G., Zhang, B., Zhang, Z., Zhao, P., Zhao, Y., Zhou, W., Zimmermann, J., Ernst, J., Raj, K., and Horvath, S. (2023). Universal DNA methylation age across mammalian tissues. *Nature Aging*, 3(9):1144–1166.

Newman, A. M., Liu, C. L., Green, M. R., Gentles, A. J., Feng, W., Xu, Y., Hoang, C. D., Diehn, M., and Alizadeh, A. A. (2015). Robust enumeration of cell subsets from tissue expression profiles. *Nature Methods*, 12(5):453–457.

Rosel, P. and Block, B. (1996). Mitochondrial control region variability and global population structure in the swordfish, *Xiphias gladius*. *Marine Biology*, 125(1):11–22.

Teschendorff, A. E. and Relton, C. L. (2018). Statistical and integrative system-level analysis of DNA methylation data. *Nature Reviews Genetics*, 19(3):129–147.

Teschendorff, A. E. and Zheng, S. C. (2017). Cell-type deconvolution in epigenome-wide association studies: a review and recommendations. *Epigenomics*, 9(5):757–768.

Zhang, H., Cai, R., Dai, J., and Sun, W. (2021). Emeth: an em algorithm for cell type decomposition based on DNA methylation data. *Scientific Reports*, 11(1):5717.

Zhou, W., Triche, T. J., Laird, P. W., and Shen, H. (2018). SeSAMe: reducing artifactual detection of DNA methylation by Infinium BeadChips in genomic deletions. *Nucleic Acids Research*, 46(20):e123.
